# Homozygous Expression of Mutant ELOVL4 Leads to Seizures and Death in a Novel Animal Model of Very Long-Chain Fatty Acid Deficiency

**DOI:** 10.1007/s12035-017-0824-8

**Published:** 2017-11-22

**Authors:** Blake R. Hopiavuori, Ferenc Deák, Joseph L. Wilkerson, Richard S. Brush, Nicole A. Rocha-Hopiavuori, Austin R. Hopiavuori, Kathryn G. Ozan, Michael T. Sullivan, Jonathan D. Wren, Constantin Georgescu, Luke Szweda, Vibhudutta Awasthi, Rheal Towner, David M. Sherry, Robert E. Anderson, Martin-Paul Agbaga

**Affiliations:** 1 Oklahoma Center for Neurosciences, University of Oklahoma Health Sciences Center, Oklahoma City, OK 73104 USA; 2 Dean McGee Eye Institute, University of Oklahoma Health Sciences Center, Oklahoma City, OK 73104 USA; 3 Harold Hamm Diabetes Center, University of Oklahoma Health Sciences Center, Oklahoma City, OK 73104 USA; 4 Reynolds Oklahoma Center on Aging, Department of Geriatric Medicine, University of Oklahoma Health Sciences Center, Oklahoma City, OK 73104 USA; 5 Department of Cell Biology, University of Oklahoma Health Sciences Center, Oklahoma City, OK 73104 USA; 6 Department of Ophthalmology, University of Oklahoma Health Sciences Center, Oklahoma City, OK 73104 USA; 7 Arthritis and Clinical Immunology Research Program, Oklahoma Medical Research Foundation, Oklahoma City, OK 73104 USA; 8 Department of Free Radical Biology and Aging Research Program, Oklahoma Medical Research Foundation, Oklahoma City, OK 73104 USA; 9 Department of Pharmaceutical Sciences, University of Oklahoma Health Sciences Center, Oklahoma City, OK 73104 USA; 10 Department of Advanced Magnetic Resonance Center, Oklahoma Medical Research Foundation, Oklahoma City, OK 73104 USA

**Keywords:** ELOVL4, Very long-chain saturated fatty acids, Synaptic vesicle fusion kinetics, Synaptic dysregulation, Seizure, Brain lipids

## Abstract

Lipids are essential components of the nervous system. However, the functions of very long-chain fatty acids (VLC-FA; ≥ 28 carbons) in the brain are unknown. The enzyme ELOngation of Very Long-chain fatty acids-4 (ELOVL4) catalyzes the rate-limiting step in the biosynthesis of VLC-FA (Agbaga et al., Proc Natl Acad Sci USA 105(35): 12843–12848, 2008; Logan et al., J Lipid Res 55(4): 698–708, 2014), which we identified in the brain as saturated fatty acids (VLC-SFA). Homozygous mutations in *ELOVL4* cause severe neuropathology in humans (Ozaki et al., JAMA Neurol 72(7): 797–805, 2015; Mir et al., BMC Med Genet 15: 25, 2014; Cadieux-Dion et al., JAMA Neurol 71(4): 470–475, 2014; Bourassa et al., JAMA Neurol 72(8): 942–943, 2015; Aldahmesh et al., Am J Hum Genet 89(6): 745–750, 2011) and are post-natal lethal in mice (Cameron et al., Int J Biol Sci 3(2): 111–119, 2007; Li et al., Int J Biol Sci 3(2): 120–128, 2007; McMahon et al., Molecular Vision 13: 258–272, 2007; Vasireddy et al., Hum Mol Genet 16(5): 471–482, 2007) from dehydration due to loss of VLC-SFA that comprise the skin permeability barrier. Double transgenic mice with homozygous knock-in of the Stargardt-like macular dystrophy (STDG3; 797-801_AACTT) mutation of *Elovl4* with skin-specific rescue of wild-type *Elovl4* expression (*S* ^*+*^ *Elovl4* ^*mut/mut*^ mice) develop seizures by P19 and die by P21. Electrophysiological analyses of hippocampal slices showed aberrant epileptogenic activity in *S* ^*+*^ *Elovl4* ^*mut/mut*^ mice. FM1-43 dye release studies showed that synapses made by cultured hippocampal neurons from *S* ^*+*^ *Elovl4* ^*mut/mut*^ mice exhibited accelerated synaptic release kinetics. Supplementation of VLC-SFA to cultured hippocampal neurons from mutant mice rescued defective synaptic release to wild-type rates. Together, these studies establish a critical, novel role for ELOVL4 and its VLC-SFA products in regulating synaptic release kinetics and epileptogenesis. Future studies aimed at understanding the molecular mechanisms by which VLC-SFA regulate synaptic function may provide new targets for improved seizure therapies.

**Electronic supplementary material:**

The online version of this article (10.1007/s12035-017-0824-8) contains supplementary material, which is available to authorized users.

## Introduction

Heterozygous inheritance of four different mutations in *exon 6* of the gene encoding the enzyme *ELOngation of Very Long-chain fatty acids-4* (*ELOVL4*) develop a form of macular degeneration known as Stargardt-like macular dystrophy (STGD3) [1–3]. Our group discovered that ELOVL4 is a fatty acid elongase responsible for catalyzing the rate-limiting condensation reaction in the biosynthesis of very long-chain fatty acids (VLC-FA; ≥C28) [4]. We further showed that one of the STGD3 causing mutations, a 5-bp deletion in the exon 6 of *ELOVL4* (797-801_AACTT), results in an enzymatically inactive mutant protein [5]. VLC-FA are present as components of more complex lipid molecules with tissue-specific distribution. ELOVL4 synthesizes VLC saturated fatty acids (VLC-SFA) that are incorporated into several sphingolipids that provide the epidermal water barrier in the skin [7–10] and into complex wax esters that contribute to the tear film generated by the Meibomian gland [11]. We found that the VLC-SFA, 28:0 and 30:0, are the predominant products of ELOVL4 in the brain as components of sphingolipids [12]. ELOVL4 also synthesizes VLC polyunsaturated fatty acids (VLC-PUFA) [4] as components of phosphatidylcholine (PC) that are enriched in retinal photoreceptor outer segments [13] in the retina and as components of sphingolipids in testes [12, 14–16].

ELOVL4 and its VLC-FA products are critical to the function of the central nervous system. Several mutations in the human *ELOVL4* gene that cause neurological and skin disorders have been identified [1, 2, 17–22]. Heterozygous inheritance of *ELOVL4* mutations causes STGD3, an aggressive juvenile macular degeneration, in the absence of any other central nervous system or skin phenotypes [1–3]. Heterozygous inheritance of other *ELOVL4* mutations causes autosomal dominant type 34 spinocerebellar ataxia (SCA34) and/or erythrokeratodermia variabilis (EKV) [17, 19, 20]; these patients show no retinal phenotype. Homozygous inheritance of *ELOVL4* mutations causes devastating neurological disorders characterized by seizures, intellectual disability, spastic quadriplegia, ichthyosis, and pre-mature death [18, 21]. Thus, ELOVL4 and its VLC-FA products play a critical, albeit unknown role in brain development and function.

ELOVL4 is poised to play a role in many parts of the developing and mature brain. ELOVL4 localizes to the endoplasmic reticulum [5, 23] and is primarily expressed by neurons in the brain, although some expression of ELOVL4 may be present in glial cells, particularly oligodendrocytes [24]. Neuronal expression of ELOVL4 is widespread but varies in a region and cell type-specific manner. High levels of ELOVL4 are found in neurons of the cerebral cortex and portions of the hippocampus [24], consistent with the seizure phenotypes associated with homozygous inheritance of mutant *ELOVL4*. In particular, neurons in the CA3 region of the hippocampus, a structure intimately involved with epileptogenesis in a number of seizure disorders, show high levels of ELOVL4 [24]. Similarly, ELOVL4 is prominently expressed in the granule cells and Purkinje cells of the cerebellum [24], which is affected by the autosomal dominant mutations in ELOVL4 that cause SCA34 [17, 19, 20]. Expression of ELOVL4 in the brain is regulated at the genomic level [25]; it begins at embryonic stages and peaks shortly after birth before declining by 30 days after birth to low, but steady-state expression into adulthood [25]. The spatial and temporal regulation of ELOVl4 expression in the brain and the association of ELOVL4 mutations with varying degrees of human neural disease indicate that ELOVL4 and its VLC-FA products are critical to neuronal health and function.

In an attempt to identify the complex roles for ELOVL4 and its VLC-FA products in health and disease, our group developed a novel animal model to study the effects of ELOVL4 and VLC-FA depletion on brain function. Homozygous expression of mutations in *Elovl4* or global deletion of *Elovl4* in mice leads to their death within hours of birth due to dehydration [6–10], which until now, has prevented investigation into the function of ELOVL4 or its VLC-FA products. To circumvent this neonatal lethality, we generated skin-rescued (*S* ^*+*^) mice that express two non-functional copies of *Elovl4* containing the 5-bp deletion found in STGD3 patients (797-801_AACTT) [1, 3], but with transgenic expression of the wild-type *Elovl4* minigene in the skin under control of both the human skin-specific *KERATIN-14* and *INVOLUCRIN* promoters, to rescue the skin barrier defect. These mice (*S* ^*+*^ *Elovl4* ^*mut/mut*^) survived, but starting at post-natal day 19 (P19), developed a severe, progressive seizure phenotype that resulted in death by P21. This is consistent with the phenotype described of children with homozygous inheritance of *ELOVL4* mutations [21]. Thus, we have developed a novel animal model that recapitulates the human condition, which permits for the first time investigation of the functional role of ELOVL4 and its VLC-FA products in the brain. The studies presented herein show that the absence of functional ELOVL4 and its VLC-FA products from the brain cause synaptic dysregulation and provide evidence that VLC-SFA are able to regulate pre-synaptic vesicle fusion kinetics.

## Materials and Methods

### Transgene Construction and Generation of S^+^Elovl4^mut/mut^ and Control Mice

Three different transgenic mice were bred to generate the skin-rescued *S* ^*+*^ *Elovl4* ^*mut/mut*^ mice. These included mice with heterozygous knock-in of the mouse *Elovl4* gene containing the 5-bp (STGD3) deletion (*Elovl4* ^*wt/mut*^) and transgenic mice expressing the mouse wild-type (wt) *Elovl4* minigene under the human *involucrin* promoter, both of which have been previously described [9, 26]. We generated the third transgenic mouse line by expressing the mouse wt *Elovl4* minigene under control of the human *keratin-14* promoter *pGEM3Z* vector (kindly provided by Elaine Fuchs, PhD, Rockefeller University, New York, NY). The human *keratin-14* (*K14*) promoter drives gene expression in the basal layer of the epidermis, outer root sheath cells of hair follicles, and in stratified squamous epithelia cells of the skin [27, 28]. To generate transgenic mice expressing the wt Elovl4 under the *K14* promoter, wt mouse *Elovl4* was PCR amplified using high-quality PCR enzymes (Thermo Fisher Scientific, Carlsbad, California) and *pCMV-Elovl4* templates as previously described [4]. The PCR product was digested with Bam HI, purified, and cloned into the Bam HI site of the pGEM3Z vector containing the human K14 promoter to generate *pGEM3Z-mouse-Elovl4* transgenic construct. The final construct was sequenced to verify proper orientation and sequence integrity of *Elovl4*. The *K14-mouse-Elovl4-K14 polyA* transgene cassette was digested with Eco RI and Hind III and used for generation of transgenic mice at Duke Neurotransgenic Laboratory Core (Duke University, Durham, NC). We characterized the *TgK14-Elovl4* (*Tg* ^*K14*^ *Elovl4*) transgenic founders by PCR. Expression of wt ELOVL4 in the skin was confirmed by western blotting.

To generate the *S* ^*+*^ *Elovl4* ^*mut/mut*^ mice, we first crossed *Elovl4* ^*wt/mut*^ mice with *Tg* ^*K14*^ *Elovl4* ^*wt/wt*^ and *Tg* ^*INV*^ *Elovl4* ^*wt/wt*^ mice to generate *Tg* ^*K14*^ *Elovl4* ^*wt/mut*^ and *Tg* ^*INV*^ *Elolv4* ^*wt/mut*^ mice, respectively. The *Tg* ^*K14*^ *Elovl4* ^*wt/mut*^ and *Tg* ^*INV*^ *Elolv4* ^*wt/mut*^ were crossed to generate the skin-rescued *Tg* ^*K14-INV*^ (*S* ^*+*^) *Elovl4* ^*mut/mut*^(*S* ^*+*^ *Elovl4* ^*mut/mut*^) mice and litter mate controls used in this study. During all crosses, mice from different parents were used. To promote survival of the *S* ^*+*^ *Elovl4* ^*mut/mut*^ mice, pregnant females from the *Tg* ^*K14*^ *Elovl4* ^*wt/mut*^ and *Tg* ^*INV*^ *Elolv4* ^*wt/mut*^ lines were maintained in an Ohmeda Medical Giraffe Incubator (Laurel, MD) until they gave birth and during the nursing period. The incubator temperature was maintained at 24 °C and the humidity held at 80–90%. Studies were performed using *S* ^*+*^ *Elovl4* ^*mut/mut*^ mice and *Elovl4* ^*mut/mut*^ mice (for embryonic culture) of both sexes and WT littermate controls of both sexes.

### Animals and Husbandry

All experimental mice were bred into a C57B6 background. Mice were maintained in a pathogen-free barrier facility on a 12 h light:12 h dark daily cycle. Light intensity at cage level was ~ 150 lx. Food and water were available at all times. All animal procedures were approved by the University of Oklahoma Health Sciences Center Institutional Animal Care and Use Committee. All procedures conformed to the National Institute of Health Guide for the Care and Use of Laboratory Animals, the Association for Research in Vision and Ophthalmology Resolution on the Use of Animals in Research, and US Public Health Service guidelines.

### Immunolabeling

Brains from *S* ^*+*^ *Elovl4* ^*wt/wt*^, *S* ^*+*^ *Elovl4* ^*wt/mut*^, and *S* ^*+*^ *Elovl4* ^*mut/mut*^ mice were collected for histology and immunolabeling at post-natal days 19–21 (P19-P21, the period of seizure activity). At least five animals of each genotype were examined. Animals were euthanized by cervical dislocation followed by decapitation. The brains were removed from the skull and hemisected on an aluminum block half-submerged in liquid nitrogen. The left hemisphere of each brain was collected for biochemical analyses. The right hemisphere was embedded unfixed in optimal cutting temperature medium (OCT; Sakura Tissue Tek; VWR, West Chester, PA), frozen on the aluminum plate, and then stored at − 80 °C. Frozen sections (10 μm thickness) were prepared and collected onto Superfrost Plus slides (Fisher Scientific, Pittsburgh, PA) and stored at − 20 to − 30 °C until used.

For immunolabeling of brain tissue, cryosections were thawed and immersed in 100% methanol at − 30 °C for 20 min, rinsed in distilled water, and then rinsed in Hank’s buffered salt solution (HBSS). In some experiments, cryosections were subjected to high-temperature antigen retrieval in 10 mM citrate buffer (pH 6.0) for 30–60 min prior to rinsing in HBSS. Non-specific labeling was blocked for 2 h at room temperature using “blocker” solution (2–10% normal goat serum + 5% bovine serum albumin + 1% fish gelatin + 0.1–0.5% Triton X-100 in HBSS). Blocker was removed and a combination of primary antibodies raised in different host species was applied overnight at room temperature. Sections were rinsed in HBSS and incubated in an appropriate combination of fluorescently conjugated secondary antibodies for 60–75 min at room temperature. Sections were rinsed again and cover-slipped using Prolong Gold + DAPI (Life Technologies-Molecular Probes) to retard photobleaching.

For immunolabeling of cultured hippocampal neurons, cultured neurons grown on glass coverslips were fixed in 4% paraformaldehyde for 15–30 min at 4 °C, rinsed in HBSS, and then incubated in blocker for 45–60 min at room temperature to block non-specific labeling. Primary antibodies were applied for 4 h at room temperature or overnight at 4 °C, coverslips were rinsed, and appropriate fluorescent secondary antibodies were applied for 75 min at room temperature. Coverslips were rinsed and mounted onto glass slides using Prolong Gold with DAPI (Molecular Probes) and viewed by epifluorescence microscopy.

Specificity of labeling methods was confirmed by omitting primary antibody or substituting normal rabbit serum for primary antibody. Specimens labeled using only one primary antibody and a combination of secondary antibodies showed no bleed-through of signals between fluorescence channels.

For histological staining, frozen sections were thawed, rehydrated in HBSS, and then stained. Nissl staining was performed by applying a 1% toluidine blue solution to frozen sections of brain for 5–10 min on a hot plate at 60 °C. Sections were rinsed several times in HBSS at room temperature, cover-slipped using Prolong Gold, and observed on the microscope. Sudan Black B was prepared as a 0.3% solution in 70% ethanol, protected from light, and stored at 4 °C until used [29, 30]. A droplet of filtered Sudan Black B solution was applied to the sections on the slides, which were allowed to stand in a humidified chamber for 10 min at room temperature in the dark. Sections were then rinsed several times in HBSS at room temperature and cover-slipped using Prolong Gold and observed on the microscope.

Wide-field fluorescence and bright-field imaging were performed using an Olympus IX70 (Olympus America, Center Valley, PA) microscope fitted with a QiCAM CCD camera controlled via the QCapture software (QImaging, Surrey, BC) or, for low-magnification imaging, an Olympus MVX10 microscope fitted with an Olympus DP71 camera controlled via the CellSens software (Olympus America). Labeling patterns in fluorescence images were assessed by superimposing images of matching fields captured independently in each fluorescence channel. Low-magnification image montages were assembled using the CellSens or Photoshop software (Adobe Systems, San Jose, CA). To prepare figures, image scales were calibrated and images were imported into the Photoshop software. If necessary, brightness and contrast were adjusted uniformly across the image to highlight specific labeling.

### Primary Antibodies for Immunolabeling

Affinity-purified rabbit polyclonal anti-ELOVL4 was raised against a synthetic peptide (aa 301-312) of wild-type mouse ELOVL4 conjugated to keyhole limpet hemocyanin [4]. This antibody recognizes the WT ELOVL4 but does not recognize the mutant form of ELOVL4 associated with STDG3 [4]. Specificity of this antibody has been confirmed by western blotting and pre-adsorption previously [4]. Deletion of WT *Elovl4* eliminates immunolabeling [31, 32]. Anti-ELOVL4 was used at a dilution of 1:300 to 1:500.

### Neuron-Specific Nuclear Protein (NeuN)

Mouse monoclonal anti-NeuN (Millipore, Cat# MAB377B; clone A60) recognizes three bands at 46–48 kDa corresponding to NeuN on western blots [33, 34] and labels most post-mitotic neurons immunohistochemically. Anti-NeuN was used at a dilution of 1:500.

### Glutamic Acid Decarboxylase, 65 kDa Isoform (GAD-65)

Mouse monoclonal anti-GAD-65 (Millipore, Cat# MAB351; clone GAD-6) was raised against GAD purified from rat brain and recognizes a single band on western blots [35]. Anti-GAD-65 was used at a dilution of 1:300–1:500.

### Active Zone

Pre-synaptic active zones were identified using a rabbit polyclonal antibody raised against recombinant Rim2 (aa 1-466) and affinity purified with a peptide corresponding to aa 41-59 of mouse Piccolo that recognizes a shared epitope in Rim1, Rim2, and Piccolo (Synaptic Systems, Gottingen, Germany, Cat# 364-003).

### Vesicular Glutamate Transporter 1 (VGluT1)

Glutamatergic pre-synaptic terminals were immunolabeled using mouse monoclonal anti-VGluT1 that was raised against a fusion protein of aa 493-560 of rat VGluT1 diluted 1:100–1:200 (NeuroMab, Davis, CA. 75-066; clone N28/9).

### Synaptic Vesicle Protein 2 (SV2)

Mouse monoclonal anti-SV2 was raised against purified synaptic vesicles and used at a dilution of 1:20–1:100 (Developmental Studies Hybridoma Bank, Cat# SV2, clone SV2. [36]).

### Positron Emission Tomography (PET)

PET imaging for the measurement of ^18^F–labeled fluorodeoxyglucose (FDG) was performed by the Research Imaging Facility at the OUHSC College of Pharmacy. ^18^F-FDG was synthesized in a Biomarker Generator BG75 (Advanced Biomarker Technologies, Knoxville, TN, USA). Briefly, ~ 10 μCi/g ^18^F-FDG was delivered with a tail vein injection to anesthetized (2% isoflurane–air mixture) mice. Two hours after injection, the mice were re-anesthetized for imaging and positioned supine in a gantry of a PET-CT dual modality machine from Gamma Medica Ideas (Northridge, CA, USA). A fly-mode CT of brain was acquired before a 10-min long list-mode PET acquisition. Through the imaging period, the mice were kept anesthetized by a 2% isoflurane–air mixture. At 2.5 h post injection, blood was collected prior to euthanasia and collection of tissues. Radioactivity of the blood, heart, lung, spleen, liver, retina, and brain was measured, along with a fraction of the prepared dose for calculation of injected dose.

The acquired images were reconstructed by filtered back projection algorithm and fused with CT image to generate a composite PET-CT image using the AMIRA 3.1 software (FEI Visualization Sciences Group, Burlington, MA, USA). Composite images were used for segmentation-based drawing of a 3D region of interest surrounding the brain. No attempt was made to correct the images for attenuation.

The radioactive counts in the region of interest were determined as Ct (counts per unit volume). Ct decay was corrected to the time of injection and expressed as % injected dose per gram or per organ.

### Metabolomics

Mice were anesthetized with isoflurane and brains were harvested directly into liquid nitrogen by removal of the skull cap and rapid separation of the brain from the spinal cord using a pair of curved forceps (store at − 80 °C). In a cold room (4 °C), frozen brains were crush homogenized into powder while submerged in liquid nitrogen and stored at − 80 °C.

### Separation and Quantification of ATP, ADP, AMP, NADH, and NADPH

Homogenized brain powder was extracted in 150 mM KOH and proteins were precipitated following incubation on ice (20 min) and then pelleted by centrifugation (10 min at 16,000×*g*). Supernatant was then filtered (0.45 μm pore size) prior to analysis. Analysis: Mobile phase solvent A [100 mM KH_2_PO_4_, 1.0 mM tetrabutylammonium sulfate (TBAS, pH 6.0)]; solvent B (acetonitrile (CH_3_CN). The injection was 100 μL and the flow rate was 1.0 mL/min. Column: Eclipse Plus C18 column with 5 μm diameter beads, 4.6 × 150 mm in length (Agilent). Elution conditions: stepwise gradients of buffer A/B [(1) 100%/0%, 0 to 2.5 min; (2) 95%/5%, 2.51 to 7.5 min; (3) 85%/15%, 7.51 to 15 min; and (4) 100%/0%, 15.1 to 25 min]. Detection: ATP, ADP, and AMP absorption at 254 nm. NADH and NADPH fluorescence excitation at 340 nm and emission at 430 nm.

### Separation and Quantification of NAD^+^ and NADP^+^

Homogenized brain powder was extracted in 5% metaphosphoric acid (HPO_3_); proteins were precipitated following incubation on ice (20 min) and then pelleted by centrifugation (10 min at 16,000×*g*). Supernatant was then filtered (0.45 μm pore size) prior to analysis. Analysis: Mobile phase solvent A [100 mM KH_2_PO_4_, 1.0 mM TBAS, (pH 6.0)]; solvent B (Acetonitrile (CH_3_CN)]. The injection was 100 μL and the flow rate was 1.0 mL/min. Column: Eclipse Plus C18 column with 5 μm diameter beads, 4.6 × 150 mm in length (Agilent). Elution conditions: Stepwise gradients of buffer A/B [(1) 100%/0%, 0 to 5 min; (2) 85%/15%, 5.01 to 10 min; and (3) 100%/0%, 10.01 to 20 min].

### Magnetic Resonance Imaging (MRI)

MRI experiments were performed using a Bruker Biospec 7.0 Tesla/30 cm horizontal–bore magnet imaging system (Bruker Biospin, Ettlingen, Germany). Animals were restrained by using 1.5–2.5% isoflurane at 0.8 L/min O_2_, placed in a 72-mm quadrature volume coil for signal transmission, and a surface coil was used for signal reception. For blood-brain-barrier permeability assessment, T_1_-weighted MR images (FLASH, TR = 60 ms, TE = 5.39 ms, 70° flip angle, 256 × 256 matrix, 4 steps per acquisition, 3.5 × 3.5 cm^2^ field of view, 1 mm slice thickness; spatial resolution of 137 μm × 137 μm) were obtained pre- and post-administration of Gd-DTPA (i.v. injection via the tail vein; 0.5 mol/L, 0.5 mL/kg body weight; Magnevist, Bayer HealthCare Pharmaceuticals, Wayne, NJ) at 2–5-min intervals over a 30-min time-course. Data processing: For determining BBB permeability, MR signal intensities were measured from regions-of-interest (ROIs) in brain tissue regions using the Paravision (Bruker) software.

### Preparation of Synaptic Membranes from Baboon Brains

We used a modified version of the protocol described by VanGuilder et al. [37, 38]; see [39] for details. Briefly, five major membrane fractions were isolated along with a starting homogenate (H) from freshly dissected baboon hippocampus: P1 (nuclear), P2 (cytoskeletal), P3 (neurosynaptosomal), PSD (post-synaptic density), and SV (synaptic vesicle). Synaptic fractions were then re-suspended in Tris-HCl buffer (pH 7.4) and separated for electron microscopy, immunoblotting, or lipid analysis.

### Electron Microscopy

Samples were fixed in a mixture of 2% paraformaldehyde and 2% glutaraldehyde in 0.1 M cacodylate buffer (pH 7.3) for 2 h on ice. The fixative was carefully rinsed out with three 0.1 M cacodylate buffer washes, keeping the pellet intact. The pellet was post-fixed in 1% osmium tetroxide in 0.1 M cacodylate buffer for 90 min at room temperature, rinsed, and dehydrated through an ethanol gradient and finally propylene oxide. Pellets were infiltrated with epon-araldite resin and heat-cured. Blocks were sectioned at 100 nm and sections were mounted on 300 copper mesh grids, stained with Sato’s lead to enhance contrast, and imaged on a Hitachi H-7600 transmission electron microscope.

### Immunoblots

Western blots were performed on 12% SDS-PAGE gels using standard electrophoresis methods. Immunoblotting for ELOVL4 in wild-type, heterozygous, and mutant animals was performed on hemisected brain homogenates (10 μg protein). ELOVL4 primary antibody (1:1000) was detected using a HRP anti-host secondary antibody (1:1000). β-Actin primary antibody (1:1000) was used with an HRP anti-host secondary antibody (1:3000) to re-probe for densitometry quantification of ELOVL4 levels. Homogenized synaptic fractions isolated from baboon hippocampus were loaded (10 μg protein). Confirmation of synaptic vesicle fraction identification was performed using primary antibodies (1:4000) against VGluT1 (rabbit clone #Bc66f), VGluT2 (rabbit clone #Dcf68), and NTT4 (rabbit anti-NTT4), all of which were kindly provided by Drs. Nicolas Bazan and Jeffery Erickson (LSU, New Orleans, LA). HRP anti-host secondary antibodies were used at a concentration of 1:2000. Blots were visualized using a Kodak Imager (Kodak Inc., Rochester, NY). The Carestream imaging software (Carestream Health, Inc., Rochester, NY, USA) was used to measure protein levels. Densitometry data were analyzed using GraphPad Prism 7 (GraphPad, La Jolla, CA).

### Lipid Analysis

Total lipids from synaptic membrane fractions were extracted following the method of Bligh and Dyer [40] with modifications [41]. Fatty acid methyl esters (FAMES) were prepared, identified, and quantified as previously described [4]. Briefly, FAMES were prepared from total lipid extracts by subjecting them to strong acid hydrolysis (16.6% HCl in methanol at 85 °C overnight). Total FAMES were quantified using an Agilent Technologies 6890N gas chromatograph (GC) with a flame ionization detector (FID), using 15:0 and 17:0 as internal standards. VLC-SFA were quantified using an Agilent Technologies 7890 GC [41] with a 5975C inert XL mass spectrometer (MS) detector (Agilent Technologies). The GC-MS was operated in the electron impact (EI) single ion monitoring (SIM) mode. The 28:0 and 30:0 response values were obtained by using the *m/z* ratios 438.4 and 466.5, respectively, along with *m/z* 74.1 and 87.1. Sample concentrations were determined by comparison to external standards, using 25:0 and 27:0 as internal standards. Multivariate ANOVA with Tukey’s post hoc was used to determine statistical significance.

### Extracellular Electrophysiology

Mice were euthanized and their brains carefully removed and placed for approximately 1 min in ice-cold oxygenated artificial cerebrospinal fluid (ACSF) solution containing 126 mM NaCl, 2.5 mM KCl, 1.25 mM NaH_2_PO_4_, 2 mM MgCl_2_, 2 mM CaCl_2_, 26 mM NaHCO_3_, 10 mM glucose, 2 mM pyruvic acid, and 0.4 mM ascorbic acid (final pH 7.4). After trimming away and applying a cut to the base (20 –30° angle), the brain was fixed to an ice-cold stage and placed in a HM650V vibrating microtome (Thermo Scientific, Burlington, ON, USA) filled with cold oxygenated slicing solution containing 240 mM sucrose, 25 mM NaCl, 2.5 mM KCl, 1.25 mM NaH_2_PO_4_, 26 mM NaHCO_3_, 0.4 mM ascorbic acid, 10 mM glucose, 10 mM MgCl_2_, and 2 mM pyruvic acid (final pH 7.4). The brain was sliced horizontally and hippocampal slices with a thickness of 350 μm were collected and transferred to a recovery chamber containing oxygenated ACSF. The slices were left in this chamber at 32 °C for 30 min and then at room temperature for at least 30 min. To record, the slices were positioned on a P5002A multi-electrode array (Alpha MED Scientific Inc., Osaka, Japan). The chamber was perfused with oxygenated ACSF at a rate of 2 mL/min at 32 °C. Six hundred (600) traces of network activity were recorded, each for a 1-s duration, under physiological conditions with continuous ACSF perfusion. For high K^+^ challenge to evoke epileptiform activity, the K^+^ concentration was increased to 7.5 mM in the ACSF. Field excitatory post-synaptic potentials (fEPSPs) were generated in the dentate gyrus (DG) region of the hippocampus by stimulating downstream electrodes along the perforant pathway. Input/output curves (I/O curves) were generated by applying increasing stimulus currents from 0 to 100 μA to the pathway and recording fEPSP responses. Calculation of I/O ratio was performed by dividing amplitude of evoked fEPSP by the amplitude of fiber valley and the resulting number was log normalized for statistical comparison. See below for details of extraction, quantification, and statistical analyses.

### Data Processing

Standard testing methods, such as ANOVA with appropriate post hoc correction, Student’s *t* test, or Mann–Whitney exact test were employed when no data collection design embedding was involved.

For electrophysiology data, raw MED64 Mobius workflow files were opened and spikes were extracted in Mobius (© WitWerx Inc.). Positive and negative spike-threshold was set to + 0.021 and − 0.021 mV, respectively. Spike traces were extracted along with 1 ms of baseline before and after the spike event, without down sampling. Raw data were filtered using a Bessel highpass (2-pole) with a cutoff frequency of 1000 Hz and a DC filter with a typical spike length set to 1 ms. The resulting file containing all extracted spikes within that slice recording was then processed in Microsoft Excel using a visual basic macro that was coded and validated by us to extract each of the final parameters for statistical comparison.

The impact of genotype and K^+^ depolarization on the five variables of interest [amplitude (+), amplitude (−), inter-spike interval, frequency, and active frequency] was analyzed by fitting linear mixed effect models as implemented by the “lme” function in the R package “nlme.” The mixed-effects model extends the classical linear model (ANOVA/regression) to accommodate complex data collection design features such as nested layers and within-group correlation. The method formulation [42], computational method, and implementation of the model in R have previously been described [43]. The complex multilayer nested repeated data collection, with multiple channels/regions per slices and multiple slices per individual brains, was accounted for by proper specification of the random effect structure. Magnitude coefficient tables and *p* values for the significance of fixed effects were extracted using the tTable method, while confidence intervals were returned by the intervals function in the nlme package. In the figures, the 95% confidence intervals (CIs) for main effects and differences per region and total are represented as vertical lines, while the *p* values above the bars show the significance of interactions, which are the difference of these effects across mouse types or K^+^ treatment. When suitable, the dependent variables were pre-processed with boxcox transformation [44] to better fulfill the normality assumptions of the main statistical procedures.

Temporal variation of frequency over the 600-s observational period (see Fig. 8) was modeled and contrasted in the framework of generalized additive models [45], as implemented in the gam function of the R package mgcv. The function relies on smoothing splines for fitting the temporal curves and is able to deal with the random effects, capturing the complex data collection design as well. Spike occurrences were modeled as Poisson random events. The curve inserts in Fig. 5 depict the log of average process rates, the link functions of the Poisson family parameter, while the error bars show 95% CIs. Main effect curves (red, black) were derived with the plot_smooth function, while the differential effect (blue) was studied with the plot_diff function. The time intervals where the difference was significant, retrieved by the plot_diff function, are marked with horizontal bars at the bottom of the figure.

### Primary Embryonic Hippocampal Cultures and Supplementation

Pregnant females were euthanized by cervical dislocation and embryonic day 18.5 (E18.5) embryos were removed into Hank’s solution + 20% NuSerum (Corning). Brains were removed and both hippocampi were micro-dissected under sterile conditions in a tissue culture hood and placed in fresh ice-cold Hank’s solution + 20% NuSerum. Both hemispheres were sliced into smaller subsections and transferred to 15 mL Falcon tubes. Each sample was washed three times with ice-cold Hank’s solution + 20% NuSerum and then three times with ice-cold Hank’s solution (allowing tissue to settle to the bottom between each wash). Hippocampal cells were then digested with 1 mL of pre-warmed digestion solution [140 mM NaCl, 5 mM KCl, 7 mM Na_2_HPO_4_, 2.5 mM HEPES + trypsin, and DNAse added fresh prior to use (final pH 7.4)] at 37 °C for 10 min before mechanical dissociation with flame-tapered/silicone-coated Pasteur pipettes. Trypsin was neutralized with an equal volume ice-cold Hank’s solution + 20% NuSerum and tissue was washed three times with ice-cold Hank’s solution + 20% NuSerum and then three times with ice-cold Hank’s solution (allowing tissue to settle to the bottom between each wash). Hippocampal cells were then dissociated with flame-tapered/silicone-coated Pasteur pipettes in 1 mL ice-cold dissociation solution (12 mM MgSO_4_·7 H_2_O in Hank’s solution + DNAse added fresh prior to use). Cells were then spun down for 5 min at 1200 rpm before being re-suspended in pre-warmed plating media and counted (Biorad, Hercules, CA automated cell counter). On day in vitro 0 (DIV0), 50,000 cells were plated per coverslip in 1 mL of plating media per well. On DIV1, a 500-μL 50% media change was done, replacing with 500 μL plating media. On DIV4, a 500-μL 50% media change was performed, replacing with 500 μL plating media + 4 μM of the mitotic inhibitor cytosine-arabinoside (ARA-C). Cells were subjected to FM1-43 imaging between DIV14-17. Supplementation: To assess the effects of re-supplying VLC-SFA, 24:0 or 28:0 + 30:0 SFAs were made into sodium salts, complexed to fraction V bovine serum albumin (BSA), and delivered to neurons in freshly prepared growth media + ARA-C at a concentration of 5 μg per milliliter. Supplementation (2.5 μg of each FA) was performed as a part of the standard DIV4 half media change (described above); after 1 week of co-incubation, another half media change was performed to reduce the amount of lipid in solution, and imaging was performed between DIV14-17 as before.

### FM1-43 Dye Studies

We tested synaptic vesicle trafficking using amphipathic fluorescent styryl dyes of FM1-43 (Invitrogen, Carlsbad, CA). FM1-43 shows a robust increase of its quantum yield when incorporated into vesicles [46, 47]. This process is reversible, resulting in less free extracellular fluorescent dye in the perfusion solution. FM1-43 (4 μM) was loaded into the synaptic vesicles by depolarization for 90 s with 47 mM KCl containing Tyrode solution. After 10 min of wash with zero-Ca^2+^ Tyrode solution, neurons were stimulated with four rounds of 90 mM KCl containing Tyrode solution. Images were collected using an Olympus IX73 microscope, an Olympus 40× objective lens, and an Evolve-512 CCD camera, and analyzed by the MetaFluor imaging software (Molecular Devices, PA) as previously described [48–51]. Fluorescence intensity was corrected for background fluorescence as detected after the experiment in each selected region. To compare release kinetics, starting fluorescence values were normalized for each synaptic bouton with the pre-stimulus level set to 1.

## Results

### Depletion of VLC-SFA in the Brain Leads to Seizures and Pre-mature Lethality in Mice

Mice with global deletion (*Elovl4* ^*−/−*^) or homozygous for STGD3 mutant *Elovl4* (*Elovl4* ^*mut/mut*^; 5-bp deletion: 797-801_AACTT) die shortly after birth from dehydration due to the loss of omega-O-acyl-ceramides that contain VLC-SFA, which are essential for epidermal water barrier function [7–10]. The *S* ^*+*^ *Elovl4* ^*mut/mut*^ mice we generated were rescued from neonatal lethality, but displayed a phenotype similar in many respects to that reported in human children with inherited homozygous *ELOVL4* mutations [21]. At P19, *S* ^*+*^ *Elovl4* ^*mut/mut*^ mice developed epileptic seizures that increased in frequency until their subsequent death at P21 (Online Resource 1). Compared to their WT littermate controls, *S* ^*+*^ *Elovl4* ^*mut/mut*^ mice were developmentally delayed, with un-opened eyes and half the body weight of their heterozygous and WT littermates at P20 (Online Resource 2a), indicating an important role for ELOVL4 and its products during development. Interestingly, the wet weights of brains harvested from *S* ^*+*^ *Elovl4* ^*mut/mut*^ mice at P20 did not differ significantly from their WT littermates (Online Resource 2b).

**Fig. 1:**
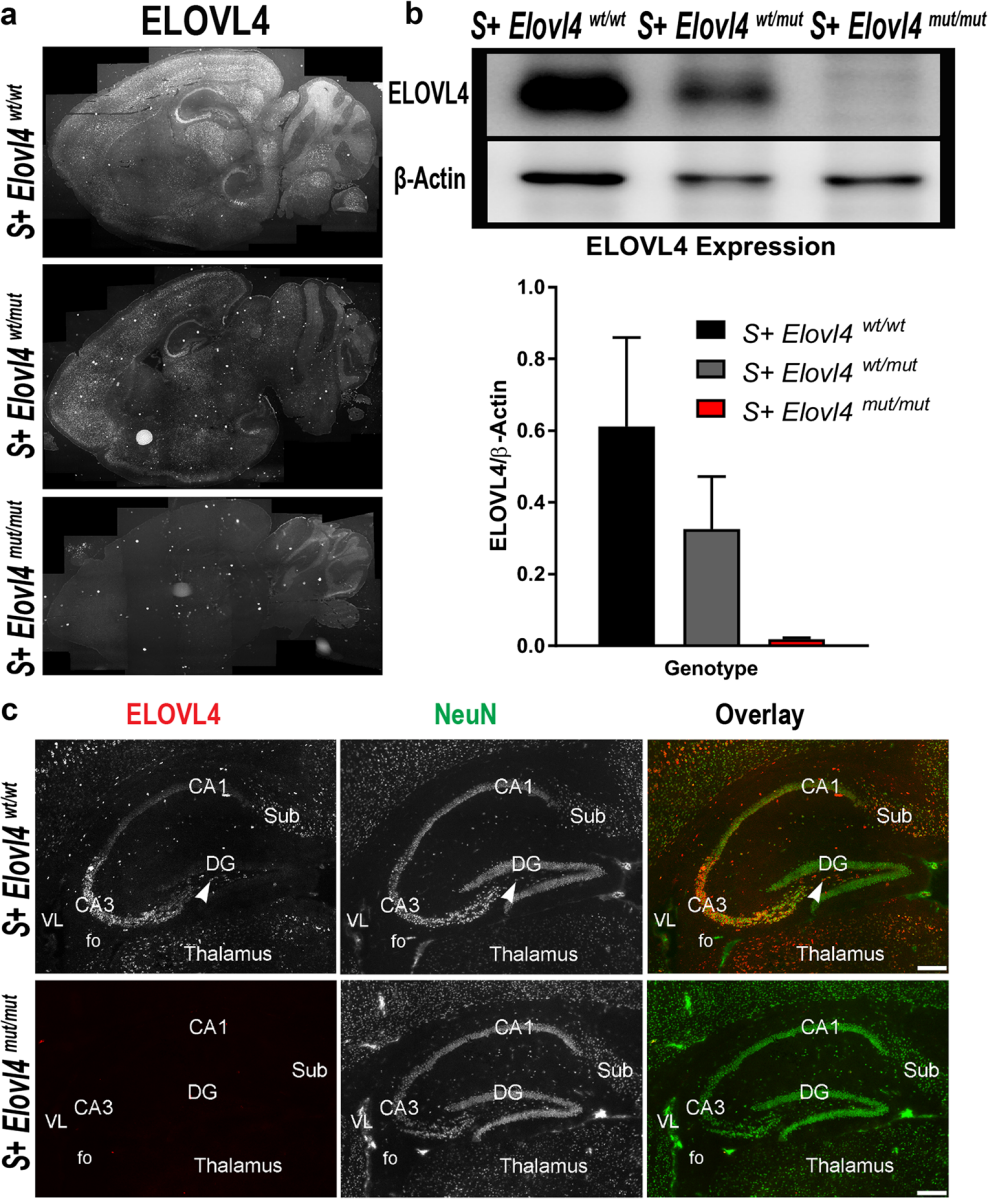
Expression of ELOVL4 in the mouse brain **a** ELOVL4 expression in *S* ^*+*^ *Elovl4* ^*wt/wt*^, *S* ^*+*^ *Elovl4* ^*wt/mut*^, and *S* ^*+*^ *Elovl4* ^*mut/mut*^ mice. **b** Western immunoblot probing for ELOVL4 in hemisected whole brain from *S* ^*+*^ *Elovl4* ^*wt/wt*^, *S* ^*+*^ *Elovl4* ^*wt/mut*^, and *S* ^*+*^ *Elovl4* ^*mut/mut*^ mice normalized to β-actin and quantified by densitometry. Statistics: one-way ANOVA with Tukey’s multiple comparisons test, ****p < 0.0001 (*n* = 6) error ± SD. **c** Distribution of ELOVL4 (red) co-localized with the neuronal nuclear marker NeuN (green) in the hippocampal formation in *S* ^*+*^ *Elovl4* ^*wt/wt*^ and *S* ^*+*^ *Elovl4* ^*mut/mut*^ mice at P20. Cornu Ammonis field 3 (CA3), polymorph layer (arrow), Cornu Ammonis field 1 (CA1), dentate gyrus (DG), subiculum (Sub), fo (fornix), VL (lateral ventricle). Scale bar = 250 μm

To determine the role of ELOVL4 in brain function, we characterized brain ELOVL4 expression by immunolabeling using a WT ELOVL4-specific antibody (Online Resource 3) that does not recognize the 5-bp del. mutant form of ELOVL4 [4]. The ELOVL4 staining pattern in brain sections from P19-P21, the period of seizure activity in *S* ^*+*^ *Elovl4* ^*mut/mut*^ mice, revealed broad but region-specific neuronal expression of ELOVL4 in the brains of *Elovl4* ^*wt/wt*^ and *Elovl4* ^*wt/mut*^ mice (Fig. 1a), as we have previously reported [24]. In contrast, *S* ^*+*^ *Elovl4* ^*mut/mut*^ mice showed no ELOVL4 labeling (Fig. 1a); residual labeling in the granule cell layer was not due to ELOVL4 as confirmed by western blotting (not shown). In *Elovl4* ^*wt/wt*^ mice, ELOVL4 expression in the hippocampal formation was very strong in neurons in CA3, subiculum, and the hilus of the dentate gyrus (DG), while neurons in CA1 and CA2 showed substantially weaker ELOVL4 labeling (Fig. 1c), as reported previously [24]. The hippocampus in *S* ^*+*^ *Elovl4* ^*wt/wt*^, *S* ^*+*^ *Elovl4* ^*wt/mut*^, and *S* ^*+*^ *Elovl4* ^*mut/mut*^ mouse brains showed normal light microscopic organization with no significant differences in size or morphology (Fig. 1c and Online Resource 4). We further confirmed loss of ELOVL4 expression in brain tissues of *S* ^*+*^ *Elovl4* ^*mut/mut*^ mice by quantitative immunoblotting (Fig. 1b).

Our preliminary studies indicated that synaptic function in the Schaffer collaterals and perforant path was dysregulated in *S* ^*+*^ *Elovl4* ^*mut/mut*^ mice. Consistent with this finding, ELOVL4 expression is high in the CA3 region of the hippocampus and the entorhinal cortex, which give rise to the Schaffer collaterals and perforant path, respectively. The hippocampus has well-established links to many different types of seizure disorders, including medial temporal lobe epilepsy [52, 53]. This is due to the hippocampus serving as a major point of connection to cortex-associated regions that allows and facilitates the spread of epileptiform activity between cortical and hippocampal networks. Finally, the hippocampus is a tractable and well-defined experimental system in which to explore neurotransmission as it relates to epileptogenesis. For these reasons, we focused our studies on synaptic structure and function in the hippocampus of the *S* ^*+*^ *Elovl4* ^*mut/mut*^ mice.

### S^+^Elovl4^mut/mut^ Mice Demonstrate Increased Energy Demand in the Brain

**Fig. 2:**
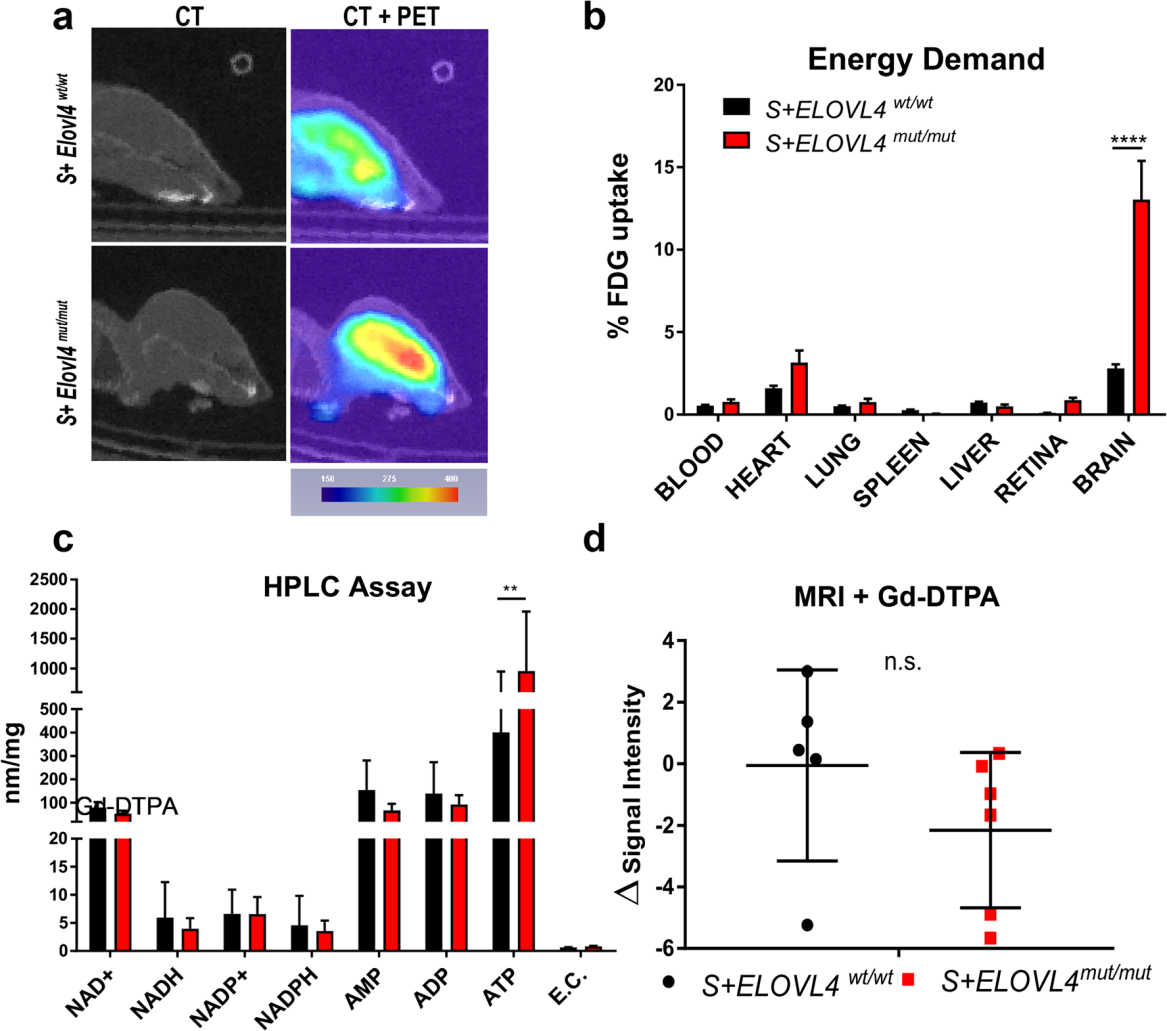
*S* ^*+*^ *ELOVL4* ^*mut/mut*^ mice demonstrate increased energy demand and ATP production. **a** Qualitative positron emission tomography (PET) imaging of *S* ^*+*^ *ELOVL4* ^*wt/wt*^ and *S* ^*+*^ *ELOVL4* ^*mut/mut*^ mice. **b** Post-mortem tissue quantification of FDG radioactivity in *S* ^*+*^ *ELOVL4* ^*wt/wt*^ and *S* ^*+*^ *ELOVL4* ^*mut/mut*^ mice. Statistics: multiple *t* tests per row, Holm-Sidak’s multiple comparisons correction, ****p < 0.0001. **c** HPLC assessment and quantification of general intermediary metabolites and energy charge (E.C.) in whole brain from *S* ^*+*^ *ELOVL4* ^*wt/wt*^ and *S* ^*+*^ *ELOVL4* ^*mut/mut*^ mice. Statistics: multiple *t* tests per row, Holm-Sidak’s multiple comparisons correction, **p < 0.01. **d** Magnetic resonance imaging with the gadolinium-based contrast agent revealed no abnormal uptake in the brains of *S* ^*+*^ *ELOVL4* ^*mut/mut*^ mice compared to wild-type controls

In view of the seizure and hyperactivity phenotype in *S* ^*+*^ *Elovl4* ^*mut/mut*^ mice, we performed PET imaging on P19-21 mice to evaluate uptake of ^18^F-FDG, an indicator of cellular glucose demand in the brain. The PET results revealed a nearly threefold increase in the amount of glucose taken up into the brains of *S* ^*+*^ *Elovl4* ^*mut/mut*^ mice compared to WT littermate controls, indicating increased metabolic demand in the brains of these seizure-prone *S* ^*+*^ *Elovl4* ^*mut/mut*^ mice (Fig. 2a). Quantification of ^18^F accumulation in the brain and other control tissues harvested post-mortem, confirmed this finding (Fig. 2b).

Increased FDG uptake can be indicative of elevated energy demand or disruption of the blood-brain barrier (BBB). We assessed both possibilities by analyzing ATP levels by HPLC and structural integrity of the BBB using magnetic resonance imaging (MRI) with Gd-DTPA, respectively. Brains of *S* ^*+*^ *Elovl4* ^*mut/mut*^ mice showed a threefold increase in ATP with no significant change in the other intermediary metabolites compared to WT littermate controls (Fig. 2c). To assess the integrity of the electron transport chain, we evaluated complex I activity using a fluorometric NADH oxidase assay. There were no differences between genotypes for complex I activity (not shown). MRI with Gd-DTPA contrast enhancement revealed no evidence for differences in BBB permeability in *S* ^*+*^ *Elovl4* ^*mut/mut*^ mice compared to WT littermate controls (Fig. 2d). Collectively, these results suggest that the increase in ATP levels may simply reflect the large energy requirement associated with seizure activity.

### VLC-SFA Are Enriched in Synaptic Vesicles

**Fig. 3:**
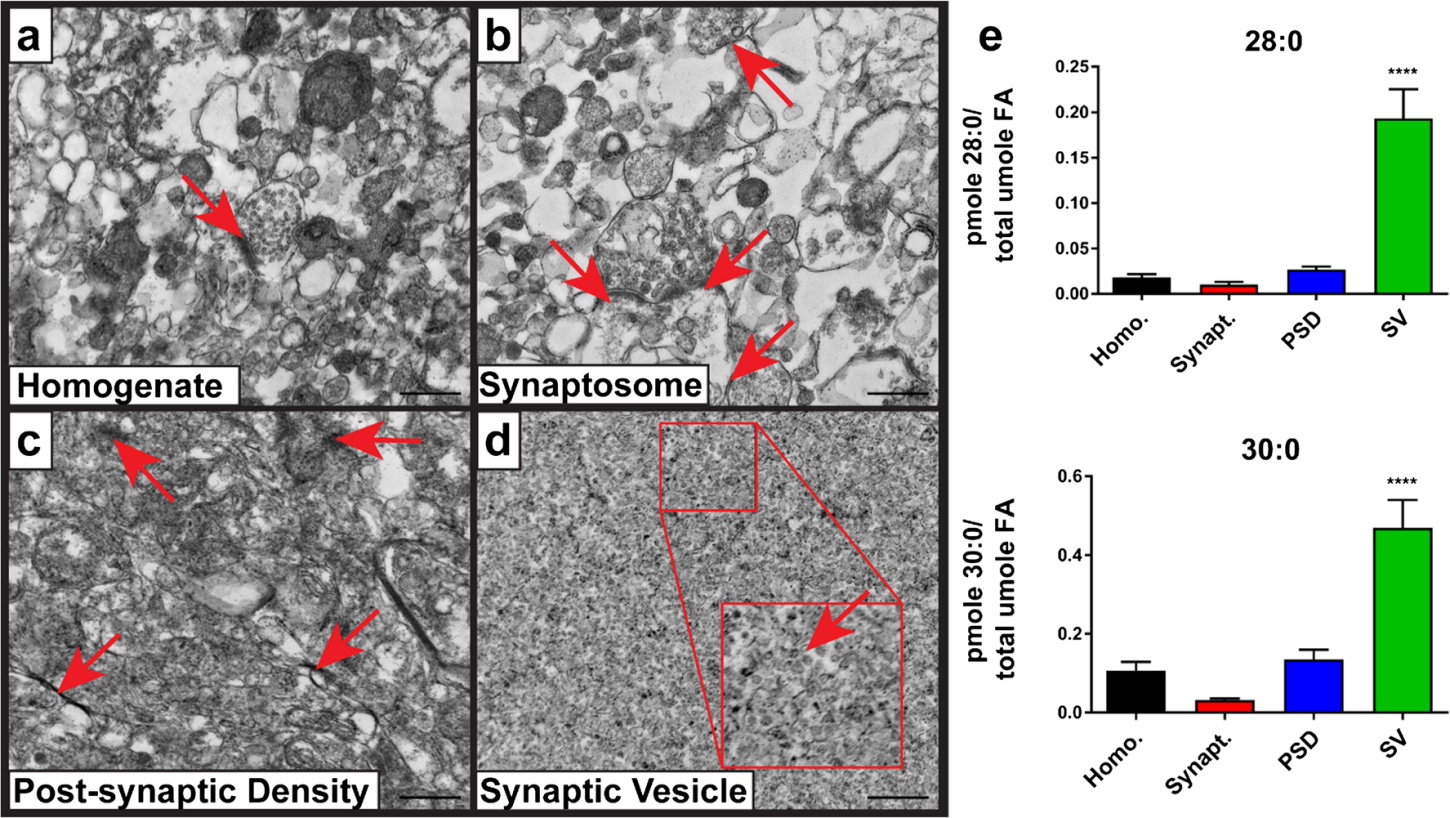
Brain-derived ELOVL4 products are 28:0 and 30:0 that are enriched in synaptic vesicle membranes. Electron micrographs of synaptic fractions isolated from baboon hippocampus by sucrose gradient centrifugation (scale bar = 500 nm). **a** Starting homogenate (Homo.) with a single neurosynaptosomal unit (arrow). **b** Neurosynaptosomal fraction (Synapt.) with multiple neurosynaptosomes in frame (arrows). **c** Post-synaptic density fraction (PSD) with multiple isolated densities indicated (arrows). **d** Synaptic vesicle fraction (SV) with high purity, vesicle indicated in zoomed inset (arrow). **e** Lipidomic analysis (GC-MS followed by GC-FID) reveals enrichment of both 28:0 and 30:0 in synaptic vesicle membranes relative to the other synaptic fractions. Statistics: two-way ANOVA with Tukey’s multiple comparison test, ****p < 0.0001 (*n* = 3) error ± SEM

To determine the effect of *Elovl4* depletion on brain lipids, we analyzed the glycerophospholipids (GPL) and sphingolipids (SPH) from hippocampus of P20 *Elovl4* ^*wt/wt*^ and *S* ^*+*^ *Elovl4* ^*mut/mut*^ mice using GC-MS and triple quadrupole tandem MS. The predominant VLC-FA in *Elovl4* ^*wt/wt*^ mouse brain were 28:0 and 30:0 VLC-SFA. To determine the subcellular localization of VLC-SFA in the hippocampus, we prepared synaptic vesicles (SV) and other fractions from freshly dissected baboon hippocampus. Baboon brains were obtained post-mortem and chosen for these studies to ensure that enough material was obtained for lipidomics following fractionation. Membrane fractions were confirmed by transmission electron microscopy (Fig. 3a–d) and western blots (Online Resource 5). Lipidomic analysis revealed that both 28:0 and 30:0 were enriched in synaptic vesicle membranes compared to other membrane fractions (Fig. 3e). Low relative levels of VLC-SFA in the starting homogenate, neurosynaptosomal, and post-synaptic density fractions reflect the low abundance of VLC-SFA-containing species relative to the other lipid species present in these fractions.

### VLC-SFA Deficiency Causes Dysregulated Pre-synaptic Vesicle Fusion

**Fig. 4:**
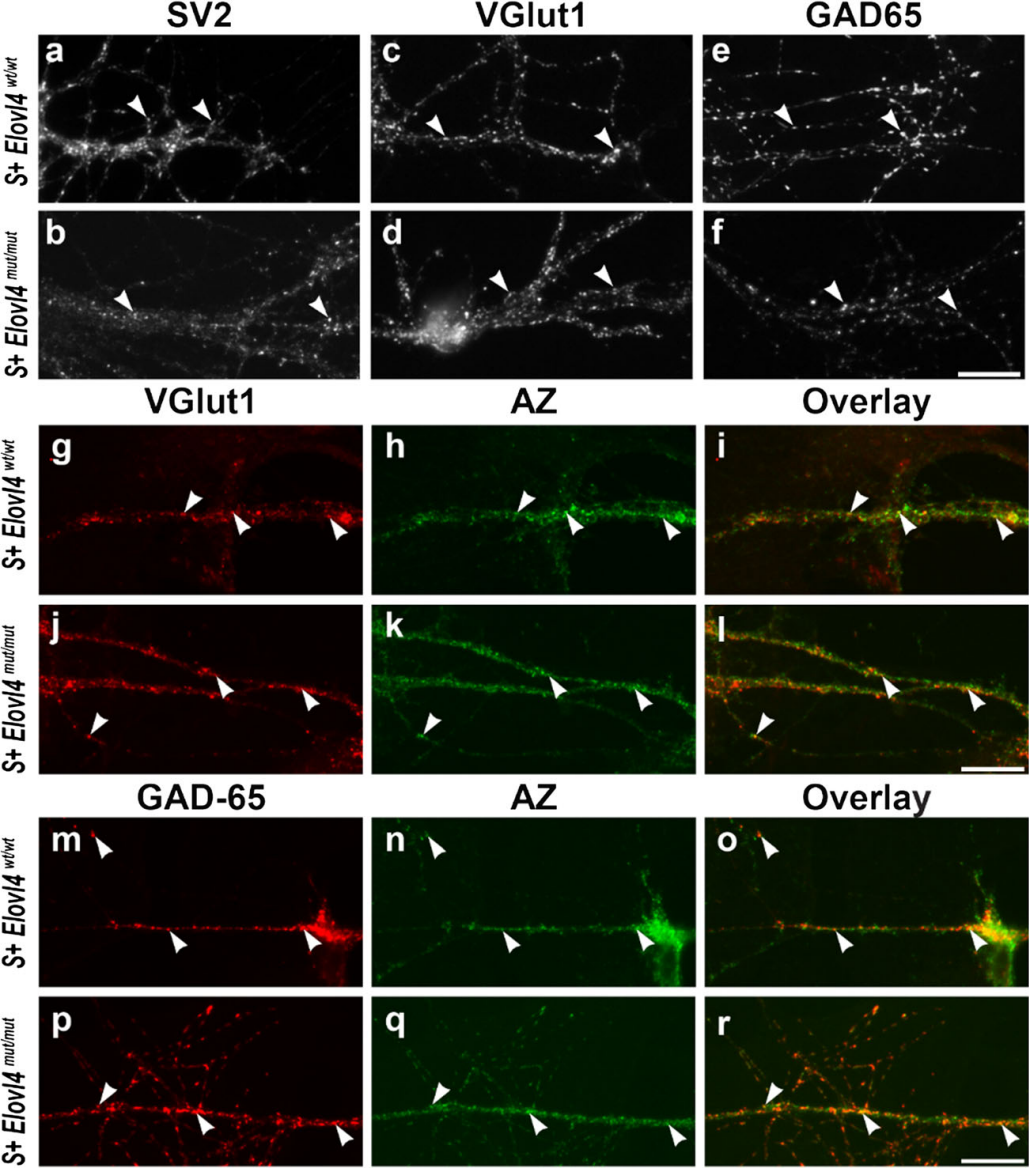
Cultured hippocampal neurons develop synapses in the absence of WT ELOVL4 with formation of both excitatory glutamatergic and inhibitory GABAergic synapses Hippocampal neurons cultured from *Elovl4* ^*wt/wt*^ and *Elovl4* ^*mut/mut*^ embryos all formed pre-synaptic terminals in vitro (arrowheads) as shown by labeling for SV2 (**a**, **b**), a ubiquitous synapse marker; VGluT1 (**c**, **d**), a marker for glutamatergic terminals; and GAD-65 (**e**, **f**), a marker for GABAergic terminals. Double labeling for VGluT1 and the pre-synaptic active zone (AZ) confirmed glutamatergic synapse formation (arrowheads) by hippocampal neurons cultured from *Elovl4* ^*wt/wt*^ and *Elovl4* ^*mut/mut*^ embryos (**g**–**l**). Double labeling for GAD-65 and the pre-synaptic active zone confirmed GABAergic synapse formation (arrowheads) by hippocampal neurons cultured from *Elovl4* ^*wt/wt*^ and *Elovl4* ^*mut/mut*^ embryos (**m**–**r**). Scale bars = 20 μm for all panels

To assess the influence of VLC-SFA on synaptic vesicle fusion kinetics, we performed FM1-43 dye studies [47, 50, 54] to measure pre-synaptic vesicle fusion rates in primary hippocampal neuronal cultures from non-skin-rescued E18.5 embryos from *Elovl4* ^*wt/wt*^ and *Elovl4* ^*mut/mut*^ mice. Hippocampal neurons cultured from *Elovl4* ^*wt/wt*^ and *Elovl4* ^*mut/mut*^ embryos formed synapses, as indicated by the presence of synaptic vesicle clusters and active zones, which are only assembled in the presence of a post-synaptic terminal, demonstrating that WT ELOVL4 is not required for synapse formation (Fig. 4). Cultured neurons of both genotypes formed glutamatergic (Fig. 4g–l) and GABAergic synapses (Fig. 4m–r). Furthermore, there were no overt differences in synapse formation between genotypes. As expected, immunolabeling confirmed that ELOVL4 was expressed in neurons cultured from *Elovl4* ^*wt/wt*^ mice, while neuronal cultures from *Elovl4* ^*mut/mut*^ mice showed no labeling for WT ELOVL4 (Online Resource 6).

**Fig. 5:**
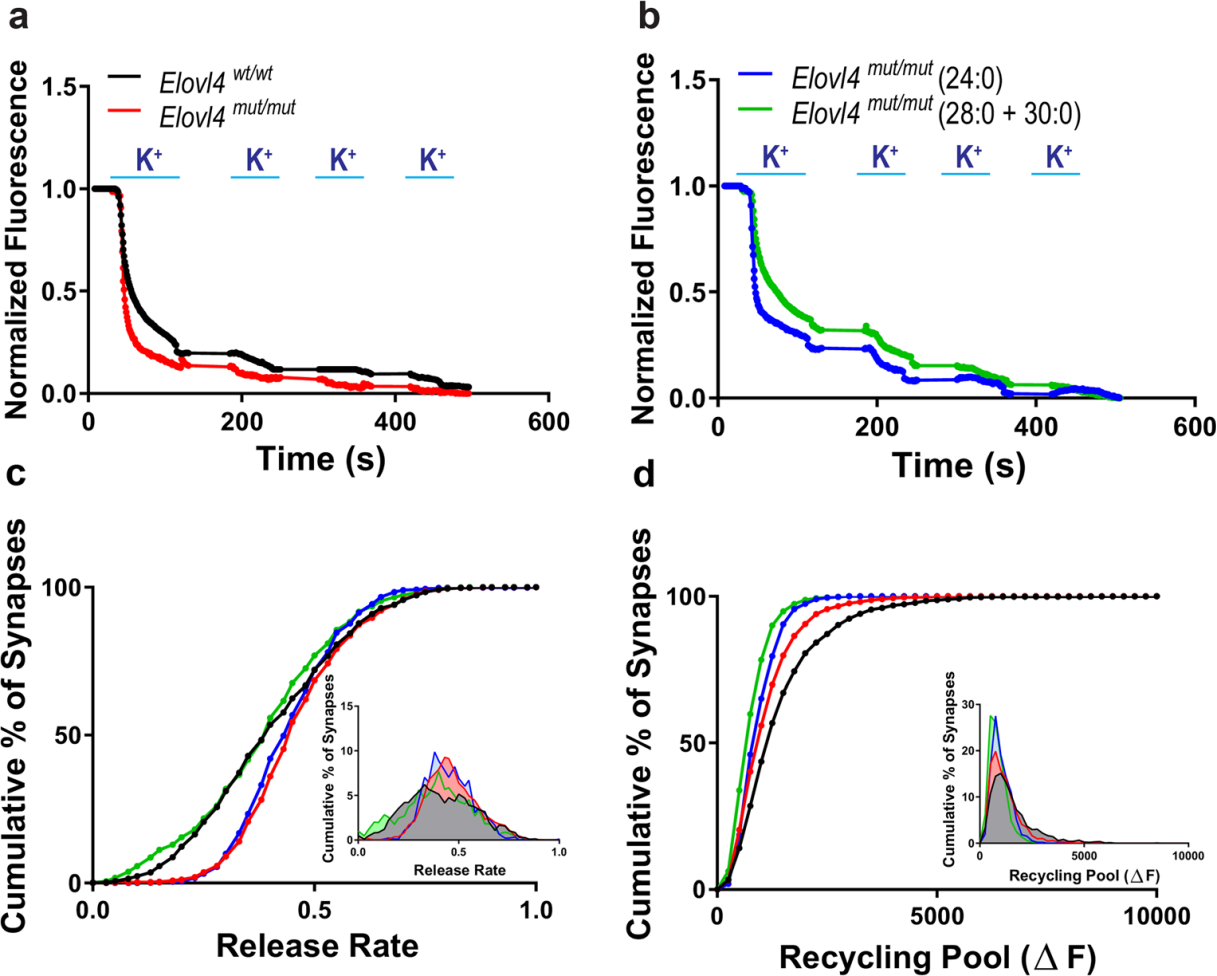
Dysregulation of synaptic vesicle release in mutant neurons lacking ELOVL4. FM1-43 fluorometric assessment of synaptic vesicle release rates and pool size in E18.5 primary hippocampal cultures collected from *Elovl4* ^*wt/wt*^ and *Elovl4* ^*mut/mut*^ embryos +/− treatment with either 28:0 + 30:0 or 24:0. **a** Representative destaining curves comparing release rates in WT (black) and mutant animals (red) in response to high K^+^ depolarization. **b** Representative destaining curves comparing release rates in mutant animals supplemented with either 24:0 (blue) or 28:0 + 30:0 (green) in response to high K^+^ depolarization. **c** Cumulative distribution of release rates for all synapses measured (Kolmogorov–Smirnov non-parametric examination of equality, *p* < 0.001). Inset: Frequency distribution of responses with slowest responding synapses falling to the left and the fastest to the right on the curve. **d** Cumulative distribution of the recycling pool of synaptic vesicles measured as total fluorescence released during the course of the experiment for all synapses measured. Inset: Frequency distribution of total fluorescent load turned over by each synapse with the synapses made up of the smallest pool at any given moment in time falling to the left, and those synapses with a larger pool at any given moment falling to the right of the curve

To test whether the enrichment of VLC-SFA in synaptic vesicle membranes affected pre-synaptic function, we performed a detailed assessment of synaptic vesicle fusion kinetics in individual synapses using FM1-43 dye [46, 47, 49–51, 54]. Loading and release of FM1-43 dye from synaptic vesicles was driven with depolarizing high K^+^ solution [50]. Analysis of synaptic vesicle fusion was performed between DIV14-17 on samples of over 2200 individual synapses per genotype (*n* = 9 cultures per genotype). Neurons from *Elovl4* ^*mut/mut*^ hippocampus showed faster pre-synaptic release of FM1-43 dye than neurons from WT control hippocampus (Fig. 5a, c). Interestingly, this shift of cumulative release frequency was not achieved by a general acceleration of all pre-synaptic neurotransmission, but rather by what appears to be selective acceleration of synaptic vesicle fusion in a subset of synapses in the absence of VLC-SFA. The fact that different synaptic populations respond to the *Elovl4* mutation differently fits with the observation that not all hippocampal neurons express ELOVL4. In addition, assessment of total fluorescence change revealed that the recycling synaptic vesicle pool was smaller in *Elovl4* ^*mut/mut*^ synapses than in WT synapses (Fig. 5d).

To distinguish any pleiotropic effects of the genetic manipulation from VLC-SFA deficiency arising from the inactivity of the mutant *Elovl4* on synaptic vesicle fusion kinetics, we supplemented neuronal cultures with an equimolar mixture of the two major ELOVL4 products in the brain (28:0 + 30:0), or with 24:0, a precursor for ELOVL4 elongation present in neurons of both genotypes, which served as a control long-chain (LC)-SFA. Assessment of *Elovl4* ^*mut/mut*^ synaptic vesicle fusion kinetics in the presence of 28:0 + 30:0 VLC-SFA (1600+ individual synapses per genotype; *n* = 10 cultures) showed rescue of synaptic release kinetics, shifting the release rate curve back to wild-type levels (Fig. 5b, c). This demonstrates that VLC-SFA regulate pre-synaptic release kinetics. Conversely, supplementation with the LC-SFA 24:0 (900+ individual synapses; *n* = 10 cultures) did not affect the release kinetics of *Elovl4* ^*mut/mut*^ synapses (Fig. 5c). Together, these results indicate that the absence of the VLC-SFA rather than the presence of the mutant STGD3 ELOVL4 protein is responsible for the pre-synaptic dysregulation observed in *Elovl4* ^*mut/mut*^ neurons. Unexpectedly, supplementation with 24:0 or 28:0 + 30:0 SFA did not rescue cumulative FM1-43 fluorescence uptake, which reflects the size of the synaptic vesicle pool (Fig. 5d). This suggests that this particular observation may be a secondary developmental or regulatory factor arising from the fact that these neurons still developed from E7.5 (onset of *Elovl4* expression) [25] until DIV4 without VLC-FA.

### *S*^*+*^*Elovl4*^*mut/mut*^ Mice Show Aberrant Neuronal Firing Patterns Under Spontaneous and Evoked Conditions

**Fig. 6:**
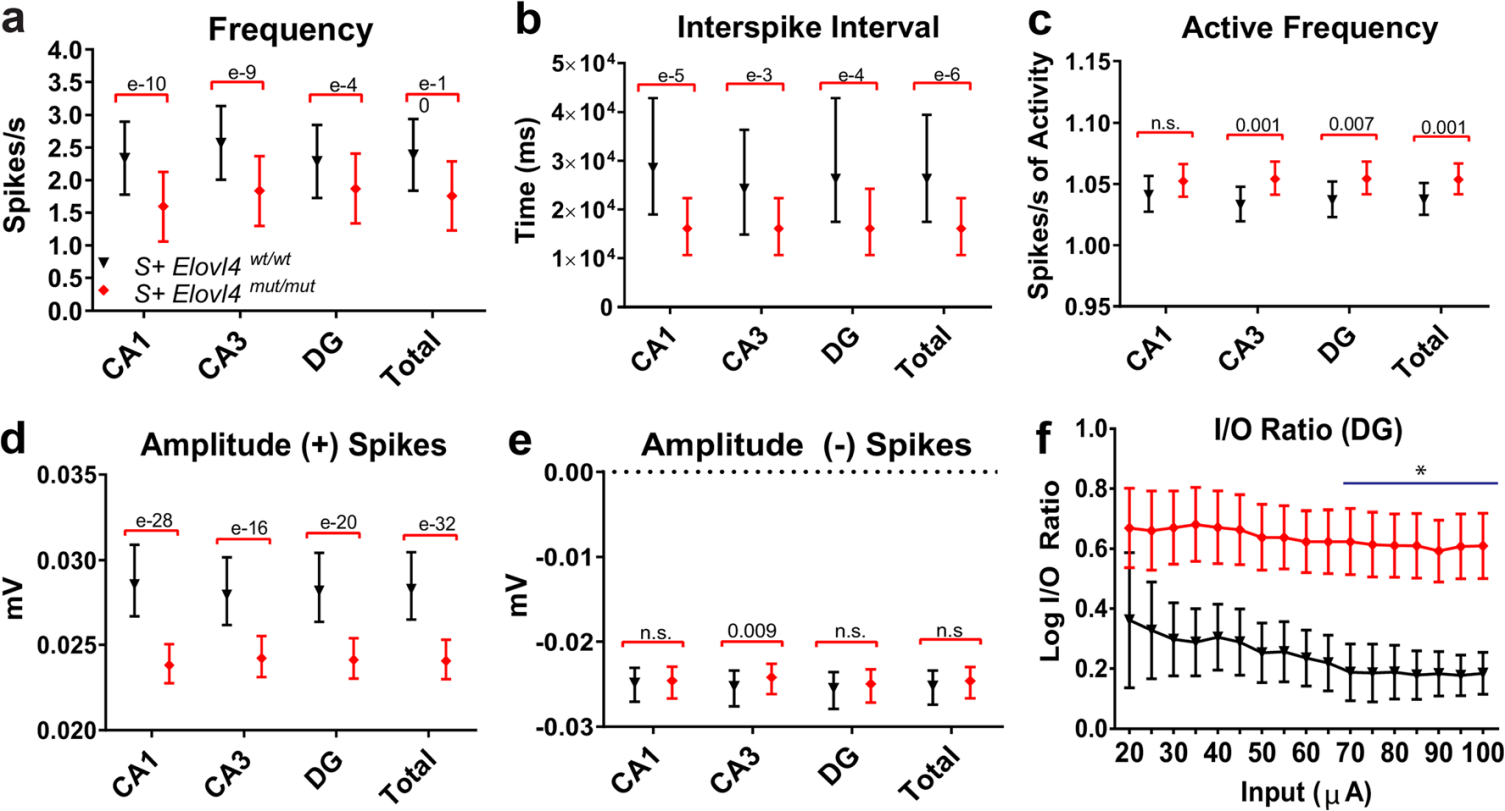
Altered hippocampal network properties and spontaneous activity in *Elovl4* ^*mut/mut*^ mice. Extracellular electrophysiology under physiological conditions in hippocampal slices ex vivo collected from *S* ^*+*^ *Elovl4* ^*wt/wt*^ and *S* ^*+*^ *Elovl4* ^*mut/mut*^ mice. The following measurements were made during 600 trace (1 s/trace) recordings of extracellular field potentials in hippocampal slices perfused with normal ACSF at 37 °C (see also suppl. Video 3). **a** Spontaneous frequency as a measure of spikes/s. **b** Spontaneous inter-spike interval (ISI) as a measure of time between spikes. **c** “Active” spontaneous frequency as a measure of spikes/s of activity. **d** Amplitude (+) spikes as a measure of spike magnitude (mV). **e** Amplitude (−) spikes as a measure of spike magnitude (mV). See methods for detailed statistics (WT: *n* = 7, slice # = 13; mut: *n* = 14, slice # = 34) error ± 95% confidence interval. **f** The input/output ratio in response to stepwise increased stimulation (20 μA minus 100 μA). Note logarithmic normalization on y-axis (statistics: two-way RM ANOVA, *p < 0.05 from 70 to 100 μA, error ± 95% confidence interval. WT: *n* = 3; mut: *n* = 10)

To better understand the epileptiform activity in the brains of *S* ^*+*^ *Elovl4* ^*mut/mut*^ mice, we performed spontaneous and evoked extracellular hippocampal field recordings. Using a 64 channel multi-electrode array, we assessed activity in the hippocampus as a whole as well as within specific sub-regions (DG, CA3, and CA1). Field potential recordings (600 consecutive 1 s traces) were performed in hippocampal slices and extracted spikes were analyzed for frequency, inter-spike interval, and amplitude to assess network activity (Fig. 6).

Under normal artificial cerebral spinal fluid (ACSF) conditions (2.5 mM K^+^), recording of spontaneous extracellular hippocampal field potentials confirmed a burst-like firing pattern in slices from *S* ^*+*^ *Elovl4* ^*mut/mut*^ animals distinct from the more sporadic, tonic firing patterns observed in WT littermate controls. In two cases, slices from *S* ^*+*^ *Elovl4* ^*mut/mut*^ mice displayed spontaneous epileptiform activity (Online Resources 7 and 8). However, *S* ^*+*^ *Elovl4* ^*mut/mut*^ hippocampal slices showed decreased average frequency of spontaneous network events overall compared to WT control slices (Fig. 6a). This bursting activity could initiate spontaneous spreading epileptiform activity throughout the whole hippocampus under physiologic conditions. Indeed, the inter-spike interval in *S* ^*+*^ *Elovl4* ^*mut/mut*^ slices was significantly reduced compared to WT controls, demonstrating a shift from a variable sporadic activity pattern in control slices to a predominately burst-like response in the mutant animals (Fig. 6b). To further analyze the pattern of responses in *S* ^*+*^ *Elovl4* ^*mut/mut*^ mice, we calculated the spontaneous frequency of activity, for which we divided the number of spikes by the duration of their activity rather than the duration of the entire recording. This measurement revealed that despite the decrease in overall frequency, when neurons in *S* ^*+*^ *Elovl4* ^*mut/mut*^ slices fire, they do so with a significantly higher frequency of activity (Fig. 6c), indicating that the hippocampus of mutant animals showed distinct periods of burst-like responses with a high frequency of activity, separated by periods of low activity or silence. The average spike amplitude under physiological conditions also was significantly lower in *S* ^*+*^ *Elovl4* ^*mut/mut*^ slices compared to WT controls (Fig. 6d, e).

**Fig. 7:**
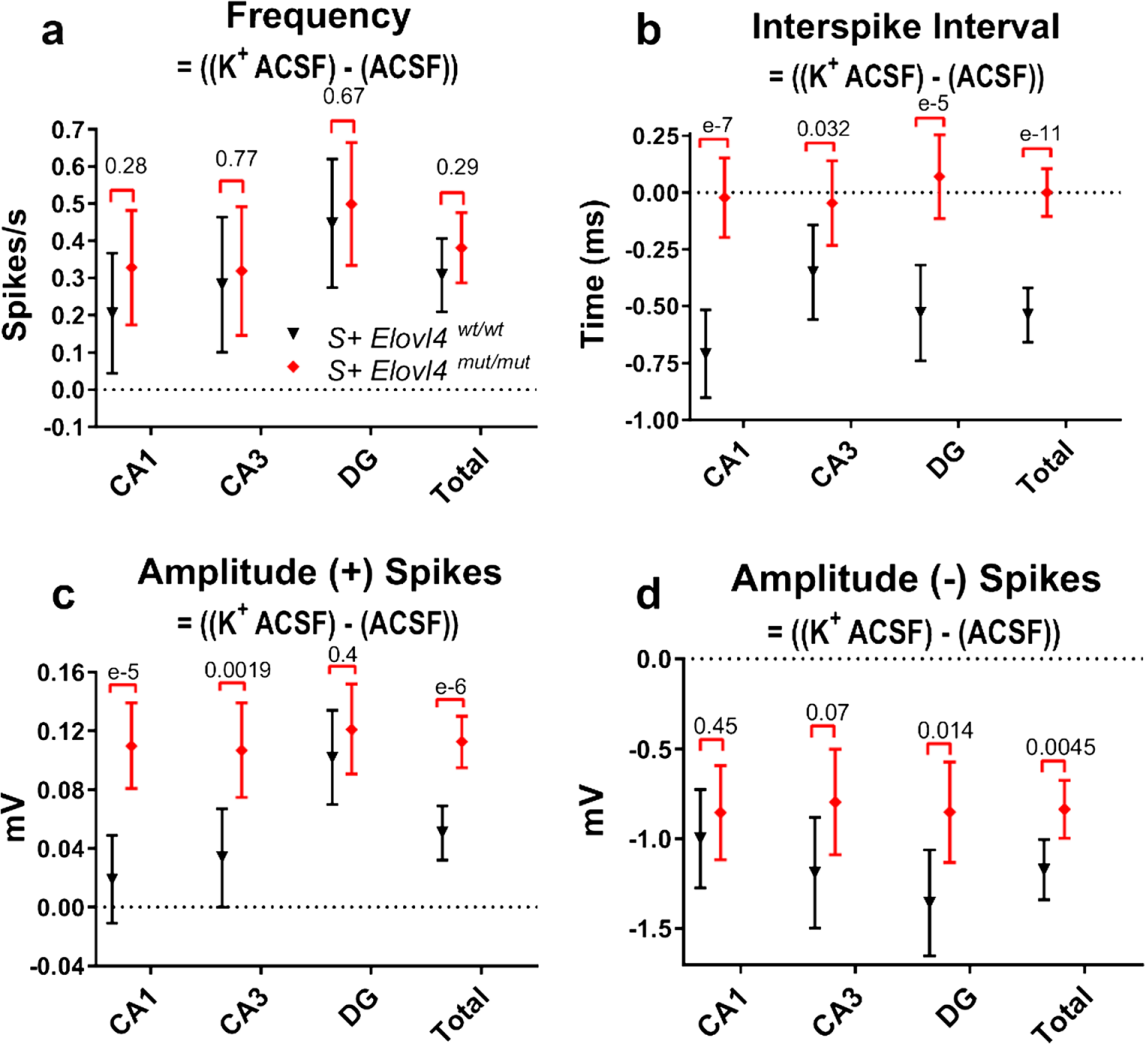
Extracellular electrophysiology under physiological conditions followed by depolarizing conditions in hippocampal slices ex vivo collected from *S* ^*+*^ *Elovl4* ^*wt/wt*^ and *S* ^*+*^ *Elovl4* ^*mut/mut*^ mice. The following measurements were made during 600 trace (1 s/trace) recordings of extracellular field potentials in hippocampal slices perfused with physiological ACSF (normal ACSF = 2.5 mM K^+^) followed immediately by a second 600 trace (1 s/trace) recording during which perfusion was switched to depolarizing, higher extracellular potassium ACSF (high K^+^ ACSF = 7.5 mM K^+^) at time = 20 s. **a** Evoked frequency presented as the difference between spikes/s at high K^+^ ACSF and spikes/s at normal K^+^. **b** Evoked inter-spike interval (ISI) presented as the time difference between spikes at high K^+^ and spikes at normal K^+^. **c** Evoked amplitude (+) spikes presented as the difference between spike magnitudes (mV) in high K^+^ and normal K^+^. **d** Evoked amplitude (−) spikes presented as the difference between spike magnitudes (mV) at high K^+^ and normal K^+^. See methods for detailed statistics (WT: *n* = 9, slice # = 22; mut: *n* = 9, slice # = 22) error ± 95% confidence interval

**Fig. 8:**
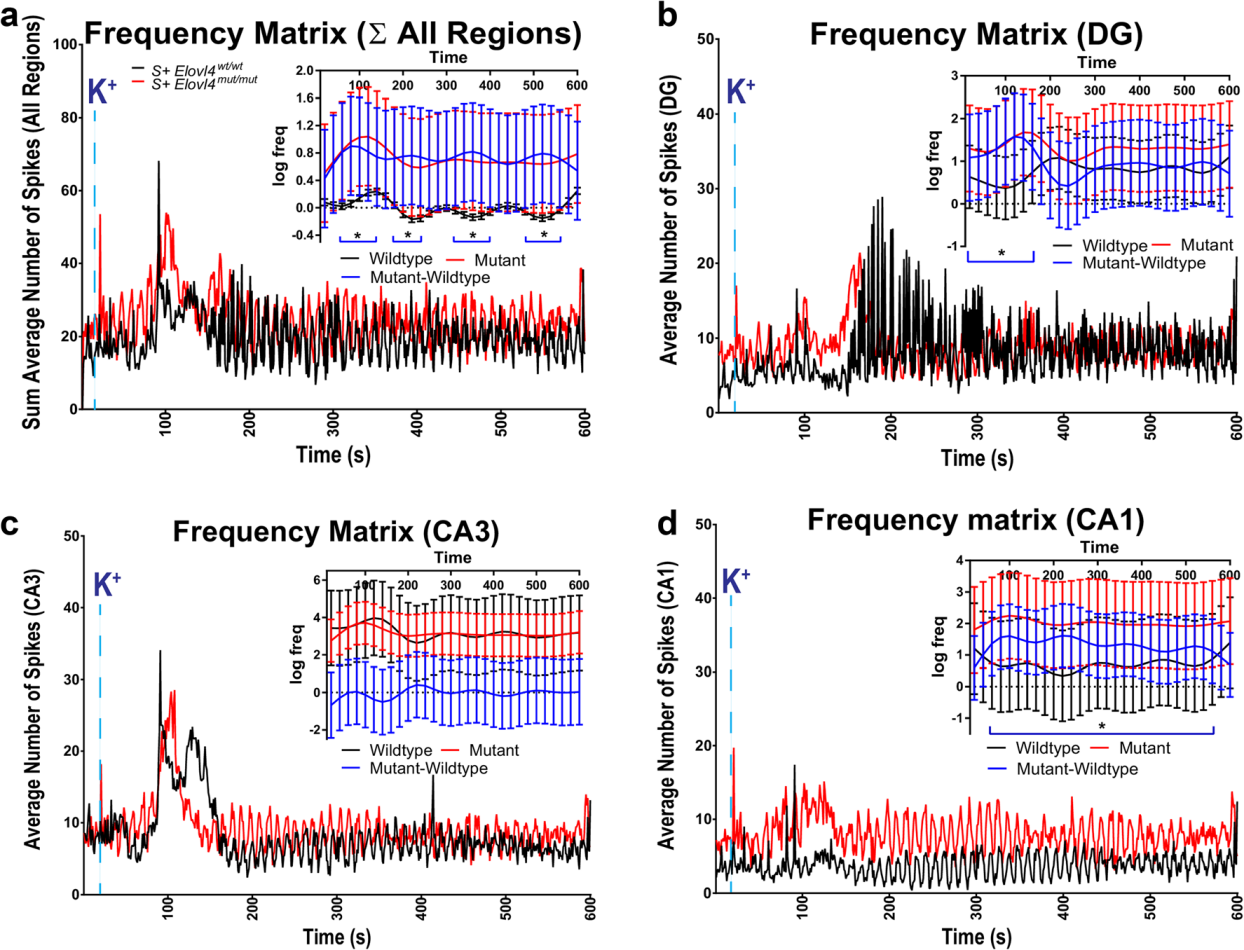
Uncontrolled spread of epileptiform activity under depolarizing condition to CA1 hippocampal region in *Elovl4* ^*mut/mut*^ mice. **a** Frequency matrix (all 64 channels × 600 s) as total spikes/s/channel (high K^+^ ACSF perfusion begins at *t* = 20 s). Inset: Sum of all regions. Spline smoothed curves of log frequency with 95% confidence bands; intervals over which the difference is significant are marked by a blue bar below. **b** Frequency matrix (all DG channels × 600 s) as total spikes/s/DG channel (high K^+^ ACSF perfusion begins at *t* = 20 s). Inset: DG region. Spline smoothed curves of log frequency with 95% confidence bands; intervals over which the difference is significant are marked by a blue bar below. **c** Frequency matrix (all CA3 channels × 600 s) as total spikes/second/CA3 channel (high K^+^ ACSF perfusion begins at *t* = 20 s). Inset: CA3 region. Spline smoothed curves of log frequency with 95% confidence bands; intervals over which the difference is significant are marked by a blue bar below. **d** Frequency matrix (all CA1 channels × 600 s) as total spikes/s/CA1 channel (high K^+^ ACSF perfusion begins at *t* = 20 s). Inset: CA1 region. Spline smoothed curves of log frequency with 95% confidence bands; intervals over which the difference is significant are marked by a blue bar below. See methods for detailed statistics (WT: *n* = 9, slice # = 22; mut: *n* = 9, slice # = 22) error (insets) ± 95% confidence interval

Given the observation of spontaneous epileptiform activity in mutant slices (Online Resource 8), we tested whether driving activity in mutant slices using a depolarizing stimulus evoked similarly enhanced neuronal activity compared to controls, by artificially elevating the probability of release. First, we compared the evoked synaptic responses following electrode stimulation in DG, which receives the main input from temporal cortex via the perforant path. We recorded excitatory post-synaptic field potentials (fEPSPs) evoked by electrode stimulation using a series of stepwise increased stimuli up to 100 μA. Synaptic I/O ratios showed significantly enhanced synaptic transmission in slices from *S* ^*+*^ *Elovl4* ^*mut/mut*^ mice compared to WT littermate controls (Fig. 6f). Second, we induced global, synchronized depolarization using ACSF with high potassium (7.5 mM K^+^) (Figs. 7 and 8) and repeated the multi-electrode array recordings. Field recordings obtained under physiological ACSF conditions and under depolarizing high K^+^ conditions showed a dramatic difference in network activation between *S* ^*+*^ *Elovl4* ^*mut/mut*^ slices and WT control slices. As expected, under depolarizing conditions, *S* ^*+*^ *Elovl4* ^*mut/mut*^ slices showed strong activation, with higher evoked spike frequency than WT controls (Fig. 7a). The inter-spike interval (ISI) in *S* ^*+*^ *Elovl4* ^*mut/mut*^ slices showed no difference between normal and high K^+^ conditions, suggesting that the mutant hippocampus was limited to burst-like responses regardless of the degree of depolarization. In contrast, WT control hippocampus showed reduced ISI in response to elevated K^+^, shifting to a more burst-like response, as expected (Fig. 7b). The average fEPSP amplitude for positive spikes under high K^+^ conditions increased in *S* ^*+*^ *Elovl4* ^*mut/mut*^ slices compared to WT control slice. Thus, when pre-synaptic release is synchronized, *S* ^*+*^ *Elovl4* ^*mut/mut*^ neurons generate a significantly larger positive fEPSP amplitude response than the WT controls and the magnitude of this difference was much larger downstream in CA3 and CA1 than it was in DG (Fig. 7c). Interestingly, the fEPSP amplitude response for negative spikes showed the opposite effect with a larger negative amplitude response in the WT control slices and in this case, the magnitude of this difference was much larger upstream in DG compared to CA3 and CA1 (Fig. 7d).

Temporal analysis of the responses to high K^+^ stimulation revealed striking differences between *S* ^*+*^ *Elovl4* ^*mut/mut*^ and WT hippocampus as a whole and among hippocampal sub-regions (Fig. 8). Onset of activity in DG (Fig. 8b) in response to high K^+^ was significantly faster in the *S* ^*+*^ *Elovl4* ^*mut/mut*^ hippocampus compared to WT control hippocampus. Despite high activity levels in the DG of WT slices, there was no subsequent downstream increase in activity in CA3 or CA1 (Fig. 8c, d). This is in stark contrast to the increased downstream activity in CA3 and CA1 of the *S* ^*+*^ *Elovl4* ^*mut/mut*^ hippocampus. These findings are consistent with the significantly higher negative spike amplitudes in DG for the WT control animals (Fig. 7d). Critically, this downregulation of activity seen in the WT control hippocampus animals was entirely absent from the *S* ^*+*^ *Elovl4* ^*mut/mut*^ hippocampus. Of particular interest, the CA1 region in the *S* ^*+*^ *Elovl4* ^*mut/mut*^ hippocampus maintained a level of high activity in response to K^+^ depolarization compared to the wt control hippocampus (Fig. 8d), consistent with the increased positive spike amplitude observed in the CA1 region of the *S* ^*+*^ *Elovl4* ^*mut/mut*^ hippocampus under high K^+^ stimulation (Fig. 7c).

## Discussion

This report establishes a double transgenic mouse model, the *S* ^*+*^ *Elovl4* ^*mut/mut*^ mouse, with homozygous knock-in of the Stargardt-like macular dystrophy (STGD3) mutation of *Elovl4* and skin-specific rescue of WT *Elovl4* expression to prevent neonatal lethality, that recapitulates critical aspects of the devastating central nervous system dysfunction associated with homozygous inheritance of *ELOVL4* mutations in humans. Critically, *S* ^*+*^ *Elovl4* ^*mut/mut*^ mice develop seizures similar to those seen in the human disease that appear by P19 and lead to death by P21. Electrophysiological analysis of hippocampal slices showed spontaneous epileptogenic activity in the *S* ^*+*^ *Elovl4* ^*mut/mut*^ hippocampus and aberrant neurotransmission through the principle circuit of the hippocampus. Dye imaging studies showed accelerated pre-synaptic release kinetics in individual synaptic terminals of cultured *S* ^*+*^ *Elovl4* ^*mut/mut*^ hippocampal neurons. Aberrant synaptic release in these cells was rescued to WT levels by supplementation of VLC-SFA via the culture medium, but not by LC-SFA. These studies establish a previously unrecognized role for ELOVL4 and its VLC-SFA products as regulators of pre-synaptic release kinetics and epileptogenesis.

A key attribute of the *S* ^*+*^ *Elovl4* ^*mut/mut*^ mouse model is that it recapitulates the severe seizure phenotype of human syndromes that arise from homozygous inheritance of mutant *ELOVL4*. Our PET and metabolic studies also revealed that the *S* ^*+*^ *Elovl4* ^*mut/mut*^ mouse brain has greatly elevated energy demands and elevated ATP levels compared to the brains of WT littermate control mice. The BBB remained intact in *S* ^*+*^ *Elovl4* ^*mut/mut*^ mice, suggesting that the increased energy demands reflect the seizures and elevated neural activity observed in these animals. Under prolonged neuronal activation, the brain will utilize the astrocyte-neuron lactate shuttle to try and sustain their energy requirement via conversion of glucose to lactate to be used as an additional energy substrate for oxidative-derived ATP production [55]. The seizures and metabolic abnormalities that characterize the *S* ^*+*^ *Elovl4* ^*mut/mut*^ brain occur in the absence of any gross defects in brain size, structure, or organization, suggesting that VLC-SFA deficiency had little effect on cell proliferation or migration in the developing brain.

In contrast, functional ELOVL4 and VLC-SFA are essential for normal synaptic function. Our studies of cultured hippocampal neurons indicate that functional ELOVL4 and its VLC-SFA products are not essential for formation of glutamatergic or GABAergic synapses. However, FM1-43 dye imaging experiments to visualize pre-synaptic release kinetics showed that synapses made by *S* ^*+*^ *Elovl4* ^*mut/mut*^ neurons exhibited accelerated kinetics from a subpopulation of synapses. Critically, re-supply of 28:0 and 30:0 via the medium rescued this synaptic release kinetics back to WT control levels, indicating that the defect arose from VLC-SFA deficiency rather than the presence of mutant ELOVL4. Furthermore, the effect on pre-synaptic release was not rescued by supplying 24:0, a precursor for ELOVL4-mediated synthesis of VLC-SFA. Thus, functional ELOVL4 is essential for VLC-SFA synthesis, consistent with previous biochemical studies [4, 5, 23]. A critical unresolved question is the identity of the specific synapses that show aberrant pre-synaptic release kinetics in the absence of VLC-SFA. CA3 pyramidal cells show the highest ELOVL4 levels in the hippocampus, which makes their synapses onto the CA1 pyramidal cells via the Shaffer collaterals excellent candidates to have aberrant release kinetics and contribute to epileptogenesis.

The aberrant synaptic release kinetics observed in the FM-dye release experiments are consistent with the spontaneous seizure activity and aberrant network responses observed in *S* ^*+*^Elovl4^mut/mut^ hippocampal slices. The striking difference in neuronal activity seen in mutant slices during normal vs. high K^+^ ACSF conditions may reflect dysregulated summation [56–59]. The observed pattern of spontaneous activity may represent an uncoupling of the neuron’s control over the timing and duration of its pre-synaptic release. Although the overall spike rate in the *S* ^*+*^Elovl4^mut/mut^ hippocampal slices under normal ACSF conditions is reduced compared to WT controls, neural activity in *S* ^*+*^Elovl4^mut/mut^ hippocampal slices in the absence of VLC-SFA shifts to a pronounced bursting activity pattern that is not observed in the WT slices. This bursting activity is a potential substrate for seizure formation, in that over time the synchronization of these bursting evens would produce highly coordinated bursts of neurotransmitter release in the *S* ^*+*^ *ELOVL4* ^*mut/mut*^ hippocampus, enhancing synaptic summation and increasing the likelihood of reaching the threshold to initiate seizure activity. This is especially true in the CA1 region of the hippocampus, which receives the largest number of pre-synaptic inputs from the ELOVL4 positive neurons in the CA3.

Mutations in the *Drosophila* ceramidase gene, *slab*, cause a loss of readily releasable vesicles as shown by FM1-43 dye studies [60]. TEM of synapses isolated from these flies revealed an increase in linked synaptic vesicles tethered at the plasma membrane, but unable to fuse. Ceramidase enzymes cleave esterified fatty acids from molecules with a sphingosine backbone [61]. In brain tissue, this class of enzymes demonstrated a significantly higher cleavage preference for longer chain saturated fatty acids [61]. Regulation of the concentration of these saturated fatty acids within SV membranes by ongoing cleavage and esterification of different length acyl chains may fine-tune pre-synaptic release; the higher the concentration of VLC-SFA, the more rigid and less fusible SV membranes may be. Confirming this concept, but using PUFA, which would impose the opposite effect (increased membrane fusion), FM1-43 dye studies revealed that the longer and more polyunsaturated the fatty acid supplemented, the more FM1-43 dye accumulated in the vesicles within the cell, indicating faster vesicle fusion and turnover [62]. These studies support the notion that synaptic vesicle membrane fatty acid composition may be an important factor in the kinetics of neurotransmitter release.

The molecular mechanism by which VLC-SFA regulates the kinetics of pre-synaptic vesicle release is currently unknown. One possible explanation is that because of their length and the absence of any *cis* double bonds, VLC-SFA could extend through the lipid bilayer and interact with fatty acyl chains esterified to glycerophospholipids and sphingolipids in the opposing bilayer. Such acyl-acyl hydrophobic interactions across the lipid bilayer could increase the van der Waals forces within the bilayer, thereby stabilizing the membranes and resisting fusion with other membranes. The absence of these interactions, which would result from the loss of functional *Elovl4* in the *S* ^*+*^ *Elovl4* ^*mut/mu*t^ mice, could increase the probability of release events such as we observed in the current study. However, follow-up studies aimed at identifying the mechanism by which the absence of VLC-SFA alters synaptic vesicle fusion kinetics will be of critical importance moving forward. Interestingly, our results suggest a role for VLC-SFA in synaptic release that is markedly different from that described for cholesterol, which facilitates synchronized evoked transmitter release [63, 64]. Such regulatory function of VLC-SFA in synaptic transmission is novel and markedly different from other lipid or lipophilic substances previously tested that acted via a receptor. For instance, platelet-activating factor (PAF) has been shown to serve a critical modulatory effect on pre-synaptic events, but all of its effects, including an influence on long-term potentiation and memory formation, were prevented by PAF receptor inhibitors [65–68].

In summary, we have identified a previously unknown role for VLC-SFA as regulators of synaptic vesicle release. These studies demonstrate that the absence of 28:0 and 30:0 causes dysregulated pre-synaptic release kinetics in hippocampal neurons by some yet-to-be determined mechanism. This disruption in synaptic transmission is one possible mechanism underlying the severe epileptiform seizures that arise from VLC-SFA deficiency. These novel findings raise further questions that must be addressed.

## Electronic Supplementary Material

ESM 1 (MP4 7262 kb)

ESM 2 (MP4 3451 kb)

ESM 3 (MP4 6717 kb)

ESM 4 (PDF 669 kb)
