# Supplementary material for: Homozygous Expression of Mutant ELOVL4 Leads to Seizures and Death in a Novel Animal Model of Very Long-Chain Fatty Acid Deficiency
Source: Mol Neurobiol. 2017 Nov 22;55(2):1795–813. doi: 10.1007/s12035-017-0824-8 (PMC5820379; doi:10.1007/s12035-017-0824-8)
Supplement: Supplementary file 4 — (PDF 669 kb) [file 12035_2017_824_MOESM4_ESM.pdf]

Homozygous expression of mutant ELOVL4 leads to seizures and death in a novel animal model of very long-chain fatty acid deficiency

Blake R. Hopiavuori[1,2], Ferenc Deák*[1,5,6], Joseph L. Wilkerson[2,4], Richard S. Brush[2,3], Nicole A. Rocha-Hopiavuori[2], Austin R. Hopiavuori[2], Kathryn G. Ozan[2], Michael T. Sullivan[2], Jonathan D. Wren[7], Constantin Georgescu[7], Luke Szweda[8], Vibhudutta Awasthi[1,9], Rheal Towner[1,10],David M. Sherry[1,4], Robert E. Anderson*[1,2,3,4,6], Martin-Paul Agbaga*[1,2,3,4,5].

[1] Oklahoma Center for Neurosciences, University of Oklahoma Health Sciences Center, Oklahoma City, OK 73104, USA
[2] Dean McGee Eye Institute, University of Oklahoma Health Sciences Center, Oklahoma City, OK 73104.
[3] Department of Ophthalmology, University of Oklahoma Health Sciences Center, Oklahoma City, OK 73104.
[4] Department of Cell Biology, University of Oklahoma Health Sciences Center, Oklahoma City, OK 73104, USA
[5] Harold Hamm Diabetes Center, University of Oklahoma Health Sciences Center, Oklahoma City, OK 73104.
[6] Reynolds Oklahoma Center on Aging, Department of Geriatric Medicine, University of Oklahoma Health Sciences Center, Oklahoma City, OK 73104.
[7] Arthritis and Clinical Immunology Research Program, Oklahoma Medical Research Foundation, Oklahoma City, OK 73104, USA
[8] Department of Free Radical Biology and Aging Research Program, Oklahoma Medical Research Foundation, Oklahoma City, OK 73104, USA
[9] Department of Pharmaceutical Sciences, University of Oklahoma Health Sciences Center, Oklahoma City, OK 73104, USA
[10] Department of Advanced Magnetic Resonance Center, Oklahoma Medical Research Foundation, Oklahoma City, OK 73104, USA

*Co-corresponding authors: Robert E. Anderson: robert-anderson@ouhsc.edu, Martin-Paul Agbaga: martin-paul-agbaga@ouhsc.edu, Ferenc Deák: ferenc-deak@ouhsc.edu

ELECTRONIC SUPPLEMENTARY MATERIAL

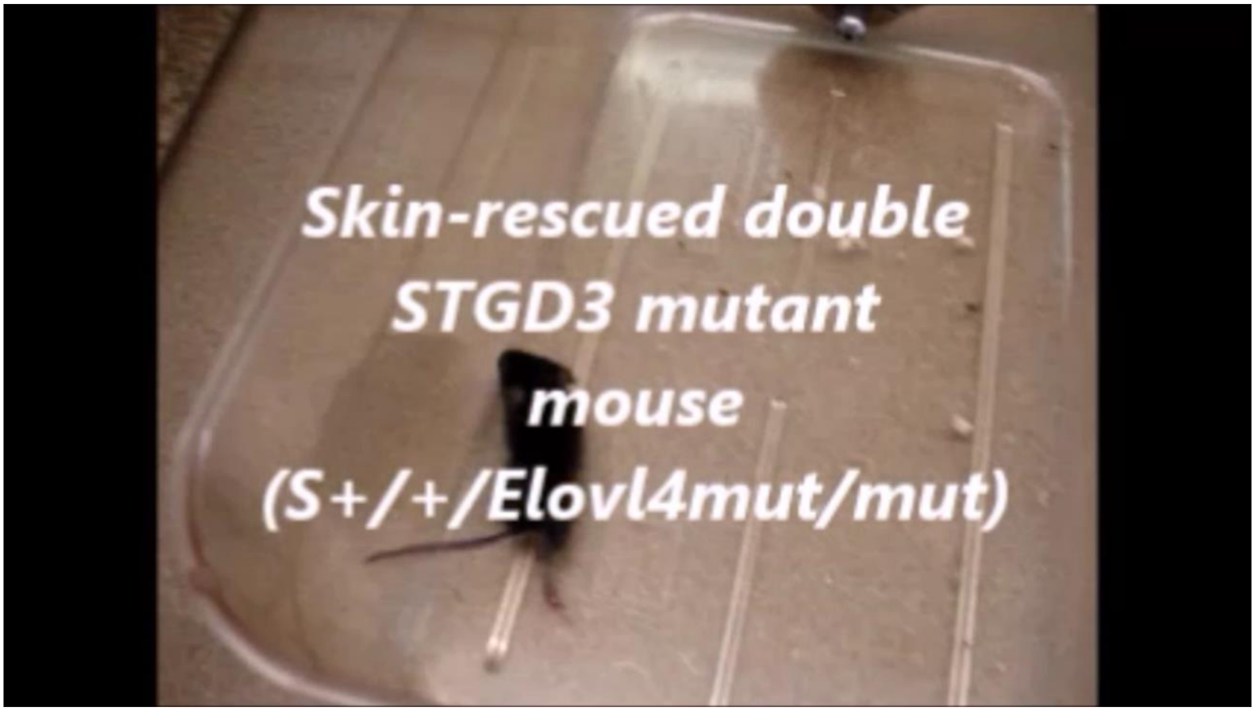

Online Resource 1. Running seizures in P19-21 S^{+}*Elovl4*$^{mut/mut}$ mouse. S^{+}*Elovl4*$^{mut/mut}$ mice develop running seizures at P19 and die two days later at P21. This cycle has not broken in the 6 years we have studied these animals. The animals run around the cage in an uncontrolled burst of motor output, before falling on their sides in a catatonic state and demonstrate respiratory difficulty during this stage of the seizure (See Video).

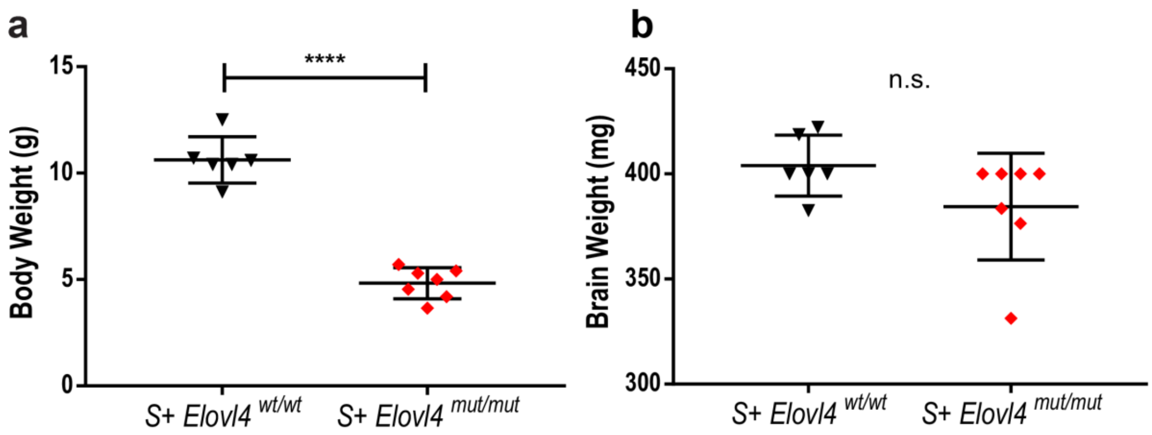

Online Resource 2. Abnormal body development in *S^{+}ELOVL4$^{mut/mut}$* mice. (a) Body weight comparison shows underdeveloped body size with developmental delay in *S^{+}ELOVL4$^{mut/mut}$* mice. (**b**) Brain size comparison shows grossly normal anatomical development. Body weight is roughly half in *S^{+}ELOVL4$^{mut/mut}$* mice compared to littermate controls, while brain weight is not significantly different. Statistics: unpaired student's t-test, **** = $p < 0.0001$.

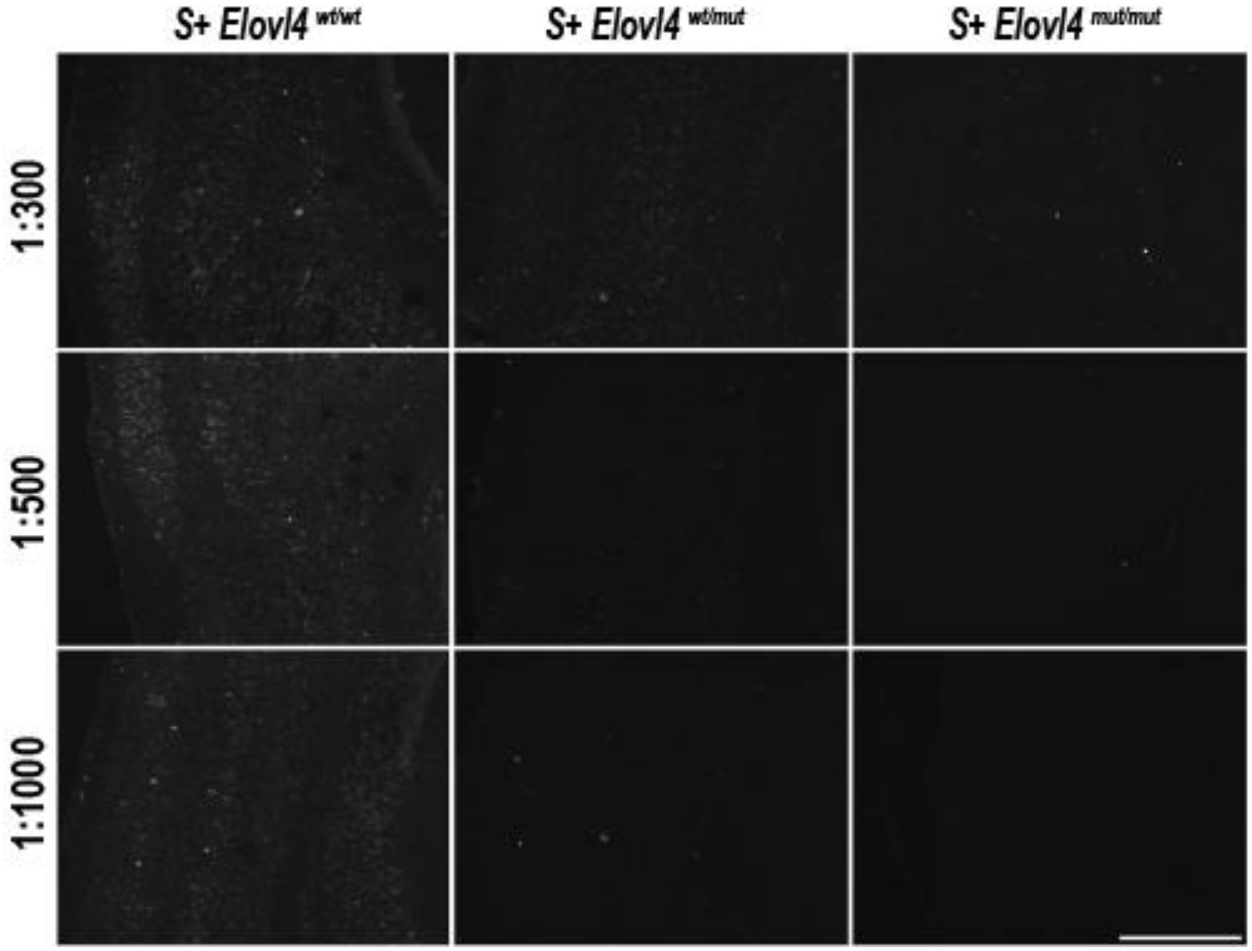

Online Resource 3. Specificity of the ELOVL4 antibody. To test the specificity of the anti-ELOVL4 antibody in brain tissue, we fluorescently immunolabeled frozen sections of brain from $S^{+}Elovl4^{wt/wt}$, $S^{+}Elovl4^{wt/mut}$, and $S^{+}Elovl4^{mut/mut}$ mice at several different antibody dilutions and examined the labeling patterns in the cerebral cortex. The ELOVL4 antibody specifically labeled comparable populations of neurons in the cortex of $S^{+}Elovl4^{wt/wt}$ and $S^{+}Elovl4^{wt/mut}$ mice, with reduced labeling intensity in $S^{+}Elovl4^{wt/mut}$ cortex, reflecting the reduced ELOVL4 expression levels associated with expression of a single wildtype *Elovl4* allele [1]. As predicted, deletion of both wildtype *Elovl4* alleles in $S^{+}Elovl4^{mut/mut}$ mice eliminated immunolabeling. These results are consistent with western blotting results showing the absence of wildtype ELOVL4 from homogenates prepared from $S^{+}Elovl4^{mut/mut}$ brain and a previous study that confirmed that the antibody did not recognize the mutant form of ELOVL4 [1]. Scale bar = 500 µm.

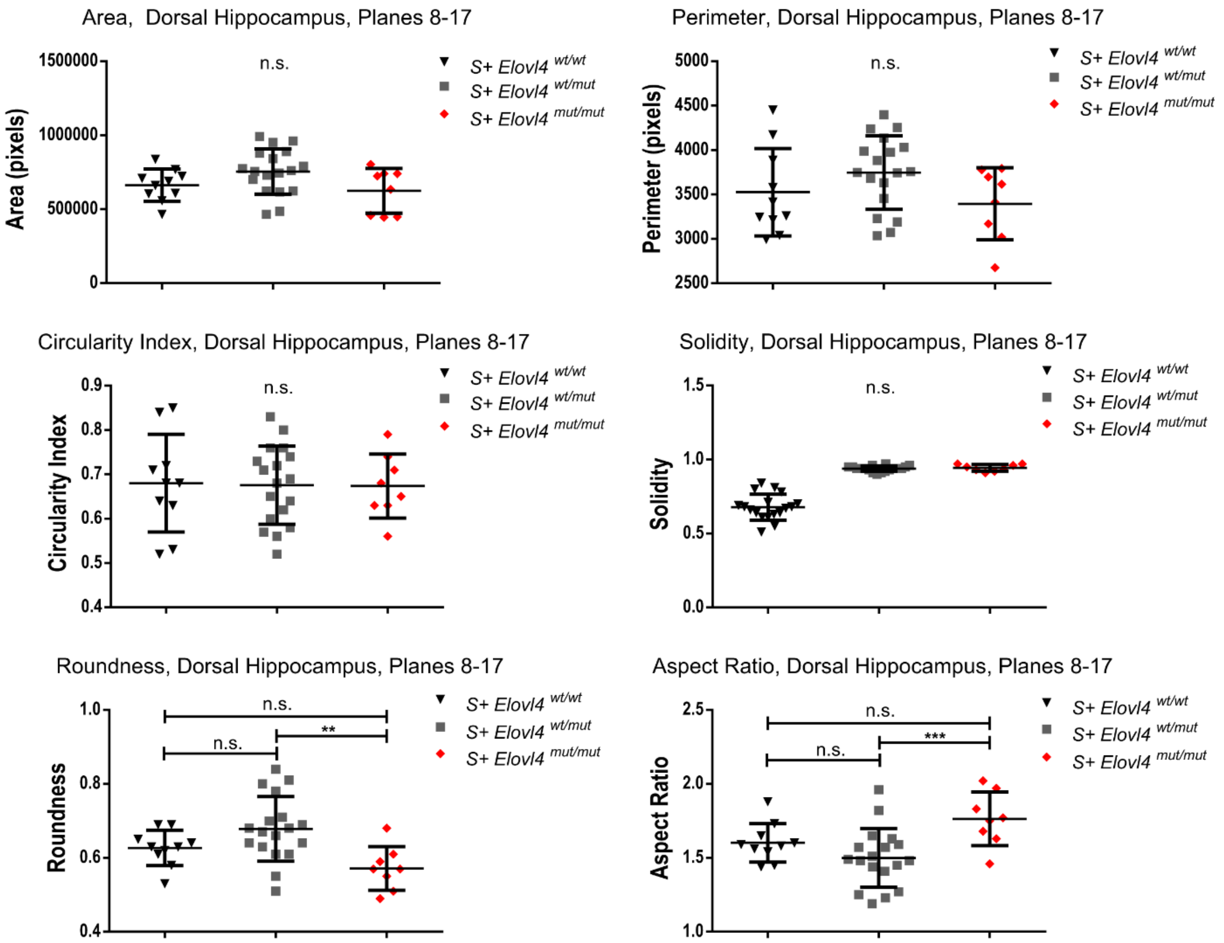

Online Resource 4. Morphometric analysis of the dorsal hippocampus of S^{+}*Elovl4*$^{wt/wt}$, S^{+}*Elovl4*$^{wt/mut}$, and S^{+}*Elovl4*$^{mut/mut}$ mice. One-way ANOVA, with Tukey's posthoc test, mean ± St. Dev. (NS, not significant; **, p<0.01; ***, p<0.001; wt: n = 10; het: n = 18; mut: n = 9).

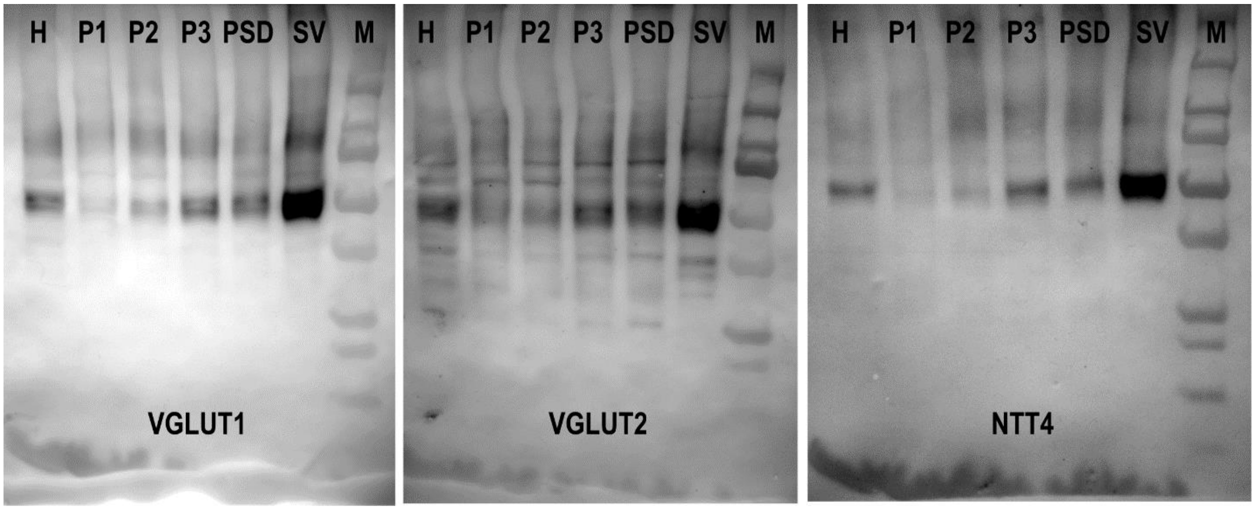

Online Resource 5. Western immunoblot validation of synaptic membrane fractions isolated from baboon hippocampus by sucrose gradient centrifugation. Synaptic vesicle fractions were validated relative to the other fractions isolated by probing with three synaptic vesicle-associated proteins: VGLUT1, VGLUT2, and NTT4. Membrane fractions are as follows: H = starting homogenate, P1 = Nuclear fraction, P2 = Cytoskeletal fraction, P3 = Neurosynaptosomal fraction, PSD = post-synaptic density fraction, SV = synaptic vesicle fraction.

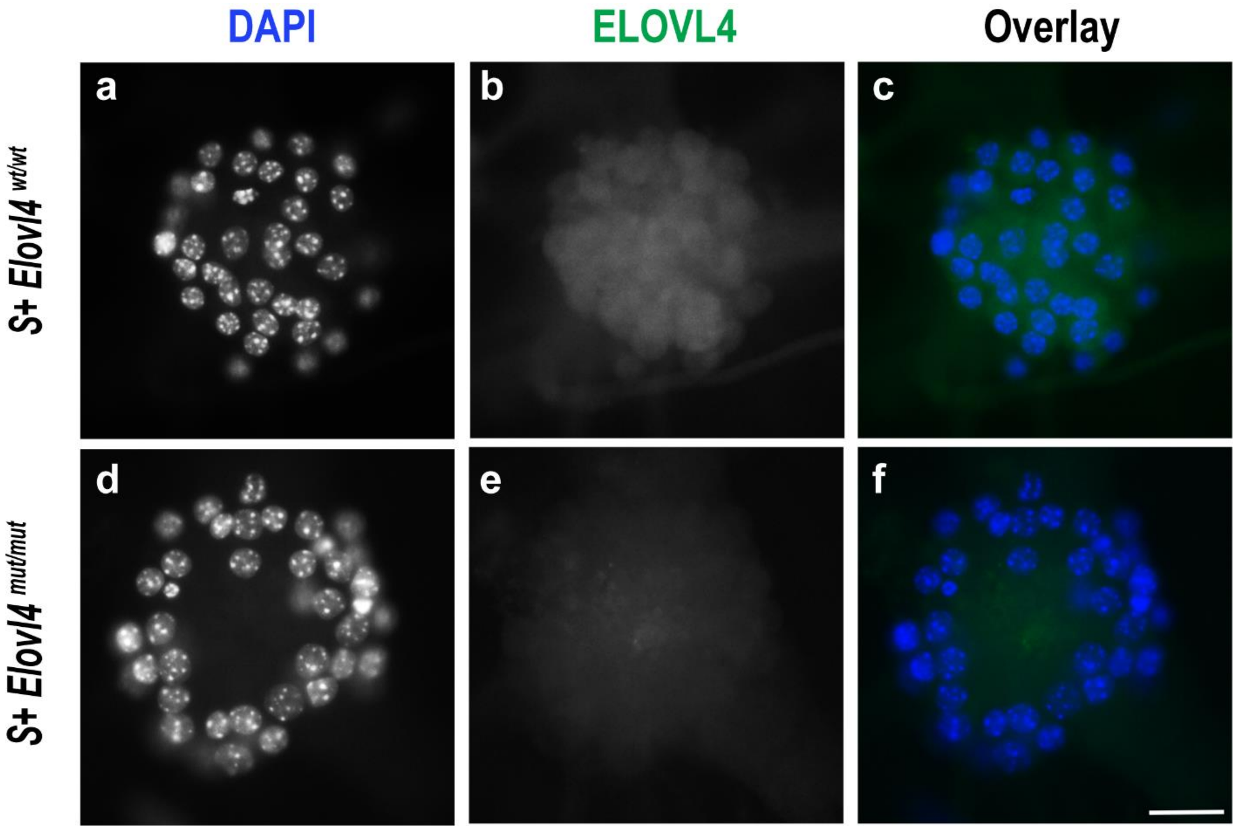

Online Resource 6. ELOVL4 expression by cultured hippocampal neurons. Clusters of cultured hippocampal neurons from *Elovl4*$^{wt/wt}$ (**a-c**) show somatic labeling for Elovl4. Neurons cultured from *Elovl4*$^{mut/mut}$ embryos (**d-f**) show no labeling for wildtype ELOVL4, as expected. Scale bar = 20 µm.

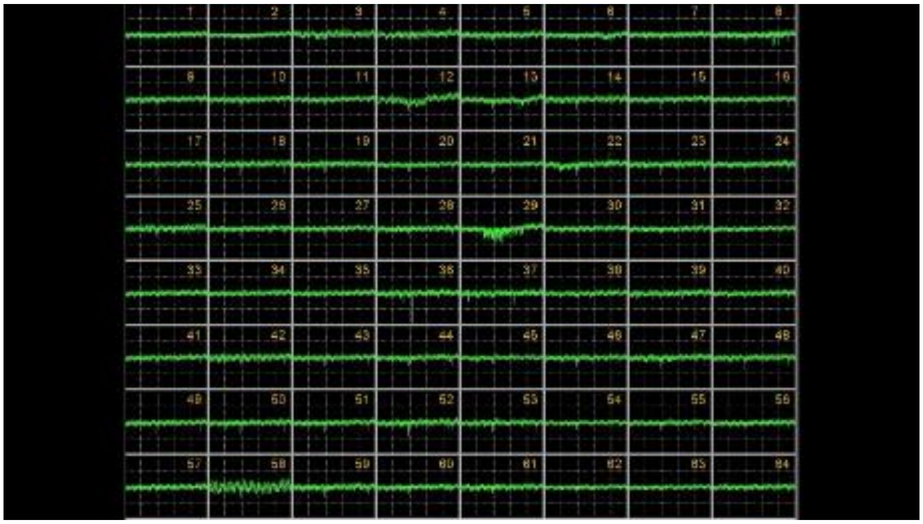

Online Resource 7. Spontaneous 64 channel extracellular recording in *S^{+}Elovl4$^{wt/wt}$* hippocampal slice. Representative extracellular slice recording of *S^{+}Elovl4$^{wt/wt}$* hippocampus shows a range of tonic and burst-like spontaneous field potentials (See Video).

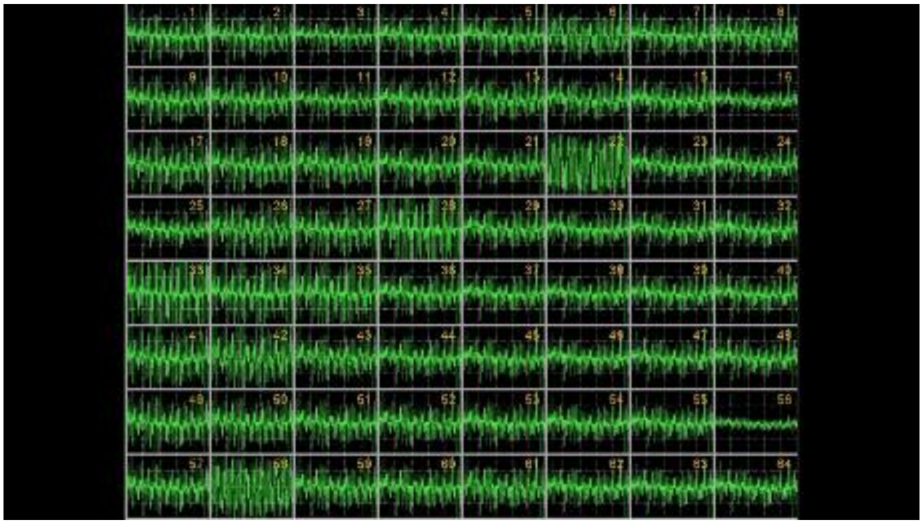

Online Resource 8. Spontaneous 64 channel extracellular recording in *S*$^{+}$*Elovl4*$^{mut/mut}$ hippocampal slice. This is an example of a spontaneous seizure event that was captured in 3 slices from our *S*$^{+}$*Elovl4*$^{mut/mut}$ cohort. There is no stimulation here. The seizure can be seen forming in the DG (lower left quadrant of the 64-channel array) and spreading quickly to CA3 (lower right quadrant of the 64-channel array) and then to CA1 (upper 3 rows of the 64-channel array). This was not typical of our *ex vivo* recordings for these mice, but is presented to demonstrate the event in the most transparent way by showing the raw recording as it happens. We emphasize that the synaptic dysregulation in these animals is capable of inducing itself into a seizure event of this magnitude in a slice without any external stimulation (See Video).
